# A novel mechanism of plasminogen activation in epithelial and mesenchymal cells

**DOI:** 10.1038/s41598-018-32433-y

**Published:** 2018-09-20

**Authors:** Moamen Bydoun, Andra Sterea, Ian C. G. Weaver, Alamelu G. Bharadwaj, David M. Waisman

**Affiliations:** 1 Department of Pathology, Halifax, Nova Scotia Canada; 2 Department of Physiology and Biophysics, Halifax, Nova Scotia Canada; 3 Department of Biochemistry and Molecular Biology, Halifax, Nova Scotia Canada; 4 Department of Psychology and Neuroscience, Halifax, Nova Scotia Canada; 5 Department of Psychiatry, Halifax, Nova Scotia Canada; 6 Brain Repair Centre, Dalhousie University, Halifax, Nova Scotia Canada

**Keywords:** Proteases, Non-small-cell lung cancer

## Abstract

Cancer dissemination is initiated by the movement of cells into the vasculature which has been reported to be triggered by EMT (epithelial to mesenchymal transition). Cellular dissemination also requires proteases that remodel the extracellular matrix. The protease, plasmin is a prominent player in matrix remodeling and invasion. Despite the contribution of both EMT and the plasminogen activation (PA) system to cell dissemination, these processes have never been functionally linked. We reveal that canonical Smad-dependent TGFβ1 signaling and FOXC2-mediated PI3K signaling in cells undergoing EMT reciprocally modulate plasminogen activation partly by regulating the plasminogen receptor, S100A10 and the plasminogen activation inhibitor, PAI-1. Plasminogen activation and plasminogen-dependent invasion were more prominent in epithelial-like cells and were partly dictated by the expression of S100A10 and PAI-1.

## Introduction

EMT is a uniquely orchestrated mechanism by which cells undergo morphological and functional changes during embryonic development (Type I), tissue repair (Type II) and cancer dissemination (Type III)^1^. EMT is characterized by progressive loss of epithelial characteristics through the deconstruction of tight junctions, reorganization of the actin cytoskeleton, loss of apical-basal polarity and gradual dissociation from the basement membrane. Eventually, cells become motile and express cytoskeletal proteins, such as vimentin and N-cadherin which enable mesenchymal movement^2^. TGFβ1, a potent inducer of EMT, binds two types of transmembrane serine/threonine kinase receptors, designated as type I and type II TGFβ receptors (TβRI and TβRII). Binding of TGFβ1 results in receptor activation and autophosphorylation which in turn phosphorylates Smad2/3 proteins^3^. Phosphorylated Smad2 and Smad3 form a complex with Smad4 which then translocates to the nucleus to induce or repress gene expression^4^.

It is generally accepted that EMT contributes to cancer cell dissemination and escape into the circulation resulting in the formation of distant-site metastasis. The latter mandates cancer cells to undergo the reverse process of MET (mesenchymal to epithelial transition) to support metastatic growth^5^. An extensive body of research has demonstrated that EMT drives cellular migration and invasiveness *in vitro* and *in vivo*^6^. Activation of the TGFβ1 signaling pathway, expression of EMT-ATFs (EMT activating transcription factors), or forced downregulation of E-cadherin resulted in increased metastasis in various cancer models. Conversely, inhibition of these processes has led to reduction in metastatic burden^7,8^. EMT has also been linked to drug resistance and stemness, both of which are hallmarks of cells of a high metastatic propensity^9^. These studies have established EMT as a keystone for successful metastasis formation in cancer.

Extracellular matrix (ECM) breakdown is a mechanism by which cancer cells escape the physical constraints of the primary tumor site. It is accomplished by the collective action of proteases such as the serine protease plasmin and matrix metalloproteinases (MMPs). Plasmin is a multifunctional protease that can degrade ECM substrates such as syndecans^10^, VCAM-1^11^, laminin and fibronectin^12^ and vitronectin^13^. Plasmin also releases matrix-sequestered growth factors like FGF-2^14^, HGF^15^ and VEGF^16^. In addition, plasmin activates pro-MMPs 1, 2, 3, 9, 13, and 14 into active MMPs^17^, and can also act as a signal-transducing ligand^18^. The diversity of plasmin functions renders the plasminogen activation (PA) system crucial in not only maintaining normal body physiology such as fibrinolysis^19^ and non-neoplastic tissue remodeling^20^ but also, when deregulated, in promoting the pathology of diseases such as cancer^21^. Plasmin is derived from its inactive zymogen plasminogen, a process that is orchestrated by a series of activators, inhibitors and receptors. Plasminogen is activated by the tissue-plasminogen activator tPA (*PLAT*) and the urokinase-plasminogen activator uPA when bound to its high-affinity receptor, uPAR. Both uPA and tPA have a limited capacity to activate plasminogen in the absence of a plasminogen receptor^22^. Binding of plasminogen to a plasminogen receptor induces a conformational change which facilitates activation by the plasminogen activators. Two major inhibitors prevent aberrant activation of plasminogen, PAI-1 which is a potent inhibitor of both tPA and uPA, and α2-antiplasmin which irreversibly inactivates plasmin^23^. uPA, uPAR, PAI-1 and several plasminogen receptors are expressed in invasive regions of various cancers including breast, colon, lung, ovary, pancreatic and prostate cancers^24–26^. Many components of the PA system are also expressed on stromal cells that support tumor growth such as uPA on myofibroblasts^25^, uPAR and the plasminogen receptor S100A10 on macrophages^24,27^ and PAI-1 on fibroblasts, macrophages and endothelial cells^24^. Higher expression levels of these proteins (particularly PAI-1) are correlated with poor cancer patient outcome^26^. The plasminogen receptor S100A10 is a member of the S100 family of proteins and its expression has been linked to enhanced plasminogen activation and invasion of cancer cells^28,29^.

It has been previously shown that EMT is often coupled with enhanced proteolytic activity particularly through the activation of MMPs. How cells undergoing EMT regulate plasminogen activation has not been addressed. In addition, the question of whether the driver of cancer cell dissemination depends on the degree to which cancer cell proteases are activated and/or the epithelial or mesenchymal state of the cell remains unanswered. Here we decipher a mechanism of regulation of plasminogen activation in both epithelial and mesenchymal cells. Our findings show that S100A10, PAI-1 and uPAR are differentially modulated in epithelial and mesenchymal cells. Surprisingly, the activation of plasminogen and subsequent invasion were partly dependent on surface levels of S100A10 and overall levels of uPAR and PAI-1 and less dependent on the mesenchymal/epithelial morphology of cells. In addition, S100A10 was shown to be regulated through canonical Smad4-dependent TGFβ1 signaling and repressed by FOXC2-mediated PI3K-mTOR signaling.

## Results

### TGFβ1 and serum withdrawal are potent inducers of epithelial and mesenchymal phenotypes in 2D *in vitro* cell models

**Figure 1:**
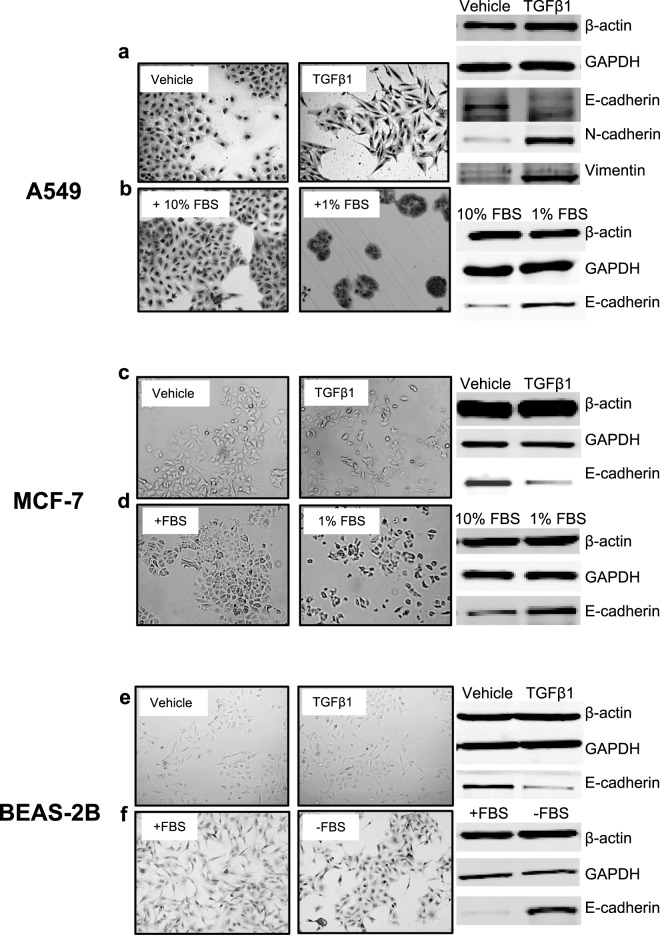
Models of epithelial and mesenchymal cells. Images of vehicle (10 mM citric acid)-treated and TGFβ1-treated (20 ng/ml for 4 days) A549 cells (**a**), MCF-7 cells (**c**) and BEAS-2B (**e**) cells. Images of A549 (**b**) and MCF-7 (**d**) cultured in the presence of 10% or 1% FBS for 4 days. Images of serum-supplemented (+10% FBS) and serum-starved (-FBS) (bottom) BEAS-2B cells (**f**) after 7 days of serum starvation. Western blot analysis of β-actin, GAPDH, E-cadherin, N-cadherin and Vimentin in the three cell model cell lines (**d–f**). N-cadherin and Vimentin were not detectable in MCF-7 and BEAS-2B cells.

To compare the regulation of plasminogen activation in epithelial and mesenchymal cells, we established three 2D *in vitro* cell models; TGFβ1-induced EMT and serum withdrawal-induced generation of epithelial-like BEAS-2B^30,31^, A549^32,33^ and MCF-7^34^ cells. Based on morphology, all three cell lines, when supplemented with 10% FBS (fetal bovine serum), appear to have an intermediate epithelial/mesenchymal phenotype (left panels; Fig. 1a–f). TGFβ1 treatment of the three cell lines induced a morphological transition into a fibroblast-like mesenchymal shape (right panels; Fig. 1a,c,e). The mesenchymal transition can be blocked by the TGFβ1 receptor inhibition (A83-01) (Supplemental Fig. 1). Notably, A83-01 treatment reverts A549 cells into a highly epithelial-like round morphology (Supplemental Fig. 1). A similar epithelial-like morphology was also achieved by culturing A549 cells^33^ in 1% FBS (Fig. 1b) and MCF-7 (Fig. 1d). Complete withdrawal of FBS from BEAS-2B cells also achieved an epithelial-like morphology (Fig. 1f) as previously described^31^. TGFβ1 induced the expression of EMT markers such as N-cadherin and vimentin and repressed E-cadherin expression in A549 cells (Fig. 1a). In contrast, serum withdrawal from all three cell lines restored E-cadherin expression (Fig. 1b,d,f). Both N-cadherin and vimentin were not detectable in BEAS-2B and MCF-7 cells as previously reported^31,35^.

### S100A10 mRNA and protein expression is regulated by SMAD4-mediated TGFβ1 signaling

**Figure 2:**
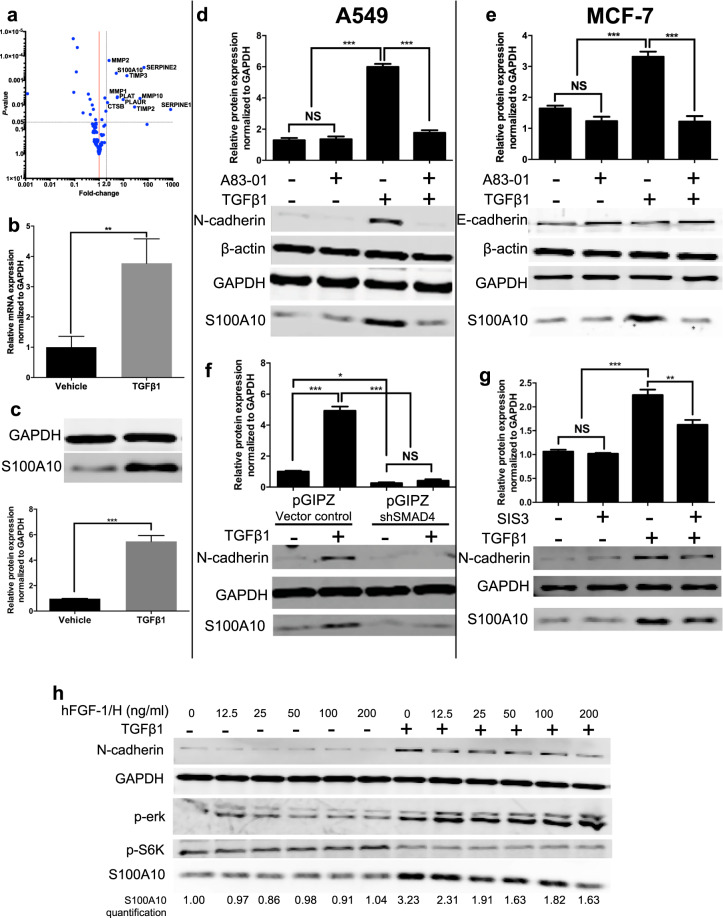
S100A10 expression is driven by canonical SMAD4-dependent TGFβ1 signaling. (**a**) Volcano plot showing differential gene expression of 130 genes involved in the protease/plasminogen activation process. The plot shows the fold-change and p-value of plasminogen activation genes in A549 cells in response to TGFβ1 (5 ng/ml) after 72 hours. RT-qPCR (**b**) and western blot analysis and quantification (**c**) of S100A10 levels in vehicle-treated and TGFβ1-treated A549 cells. Western blot analysis and quantification of S100A10 protein levels in A549 cells (**d**) and MCF-7 cells (**e**) treated with TGFβ1 with or without the TGFβR1 inhibitor (A83-01, 25 μM). Western blot analysis (**h**) and S100A10 protein quantification of TGFβ1-treated cells transfected with a stable pGIPZ shRNA knockdown construct targeting SMAD4 (**f**) or 10 μM of the SMAD3 inhibitor SIS3 (**g**). Western blot analysis and quantification of protein lysates from vehicle-treated and TGFβ1-treated A549 cells in the presence of ascending concentrations of 0 to 200 ng/ml of bhFGF-1/H after 72 hours (**h**). The gene names referred to are: PLAUR (uPAR), PLAU (uPA), SERPINE 1 (PAI-1), SERPINE 2 (PAI-2), CTSB (cathepsin B).

We first examined the expression of 130 putative extracellular protease genes relevant to the PA system (Supplemental Table 1) during TGFβ1-induced EMT in A549 cells^36^ (see methods). An overall upregulation of these genes was observed in TGFβ1-treated A549 cells indicating their potential participation in EMT. Using a *p*-value of 0.05 and a greater than two-fold difference as cut-offs, we identified 11 significantly upregulated genes (*SERPINE1 (PAI-1)*, *SERPINE2 (PAI-2)*, *TIMP2*, *MMP10*, *PLAUR (uPAR)*, *TIMP3*, *PLAT (tPA)*, *MMP1*, *S100A10*, *MMP2* and *CTSB (cathepsin B)*) (Fig. 2a). Interestingly, *S100A10* was the only plasminogen receptor to be significantly upregulated by TGFβ1 (5.06-fold increase, *p*-value = 0.0005) among all 13 characterized plasminogen receptors^22^. Plasminogen binding to cell surface receptors is a rate-limiting step in the activation of plasminogen by plasminogen activators^37^. Therefore, we further investigated S100A10 regulation in epithelial and mesenchymal cells. We first confirmed that TGFβ1 treatment increased mRNA expression of S100A10 (Fig. 2b). TGFβ1 also upregulated S100A10 protein expression (4.89-fold) in A549 cells (Fig. 2c) in a dose-dependent manner (Supplemental Fig. 2b). To exclude the possibility that the observed increases in S100A10 were limited to A549 cells, we treated multiple cancer cell types known to undergo EMT in response to TGFβ1 treatment. The upregulation of S100A10 protein by TGFβ1 was also observed in MCF-7 (Fig. 2e), HMLE^9^, Panc10.05 and BEAS-2B (Supplemental Fig. 2c) cells. Next, we utilized the compound, A83-01 to inhibit TGFβR1-mediated EMT^38^. TGFβR1 inhibition decreased N-cadherin expression and importantly abrogated S100A10 upregulation after TGFβ1 treatment in A549 and MCF-7 cells (Fig. 2d,e).

In contrast to Panc10.05 cells, BxPC3 cells harbor a homozygous deletion in Smad4 and are therefore not responsive to TGFβ1^39^. Therefore, TGFβ1 did not upregulate S100A10 in BxPC-3 cells (Supplemental Fig. 2d). This presented the possibility that Smad4 might be part of the pathway regulating S100A10. To assess the effect of canonical SMAD-dependent TGFβ1 signaling on S100A10 expression, *SMAD4* was depleted in A549 cells using short-hairpin RNA. SMAD4-depleted cells treated with TGFβ1 failed to upregulate S100A10 (Fig. 2f). Similarly, SMAD3 inhibition with the inhibitor, SIS3^40^ achieved a similar reduction in S100A10 upregulation upon TGFβ1 treatment (Fig. 2g). In addition, we also utilized bhFGF/H, which has been demonstrated to inhibit TGFβ1-induced EMT in A549 cells^41^. bhFGF/H inhibited both N-cadherin and S100A10 upregulation by TGFβ1 in a dose-dependent manner in A549 (Fig. 2h) and BEAS-2B cells (Supplemental Fig. 2e). The question of whether the S100A10 promoter or any intragenic sequences contain a SMAD binding motif is not known. We performed a TRANSFAC transcription factor analysis^42^ on the promoter sequence of S100A10 (2000bp upstream and 1000 bp downstream of transcription start site). No binding sites were detected for smad proteins in the examined DNA region (Supplemental Fig. 4) indicating that TGFβ1/Smad signaling modulates S100A10 expression through a mechanism that may not involve smad protein binding to the promoter region. Collectively, these results confirmed that the plasminogen receptor S100A10 is uniquely regulated by TGFβ1/TGFβR1/SMAD4 signaling.

### S100A10 is a TGFβ1-responsive gene and not an EMT gene

**Figure 3:**
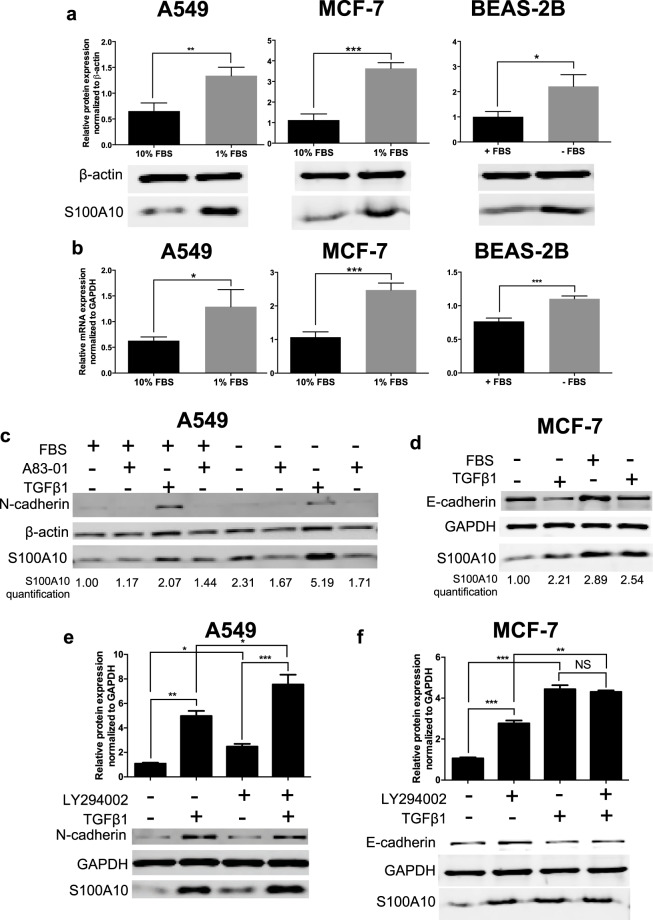
Serum starvation or PI3K inhibition has an additive effect on TGFβ1-induced increase of S100A10. Western blot analysis and quantification (**a**) and RT-qPCR (**b**) of S100A10 in serum-deprived A549, MCF-7 and BEAS-2B cells. A549 and MCF-7 cells were supplemented with 10% or 1% FBS while BEAS-2B cells were culture in the complete presence (10% FBS) or absence of serum. Western blot analysis and S100A10 protein quantification in A549 (**c**) and MCF-7 (**d**) cells treated with TGFβ1 and A83-01 for 4 days in the presence/absence of FBS. Western blot analysis and S100A10 quantification in A549 cells (**e**) and MCF-7 (**f**) treated with the PI3K inhibitor LY294002 +/− TGFβ1.

TGFβR1 inhibition or *SMAD4* depletion in A549 and MCF-7 cells treated with TGFβ1 prevented these cells from undergoing EMT hence not allowing us to discern a TGFβ1-specific response from a global EMT effect on S100A10. To address the issue of whether expression of S100A10 was dictated by cell morphology, we compared S100A10 expression by epithelial and mesenchymal cells, independent of TGFβ1, using the serum-withdrawal models (Fig. 1). Surprisingly, serum withdrawal, which induces an epithelial-like morphology, also upregulated S100A10 protein (Fig. 3a) and transcript (Fig. 3b) in A549, MCF-7 and BEAS-2B cells. Importantly, TGFβ1 treatment of serum-supplemented BEAS-2B cells, that are mesenchymal in morphology, upregulated S100A10 protein expression (Supplemental Fig. 2c). Serum withdrawal increased S100A10 expression and was exacerbated in the presence of TGFβ1 in A549 and MCF-7 cells and was abrogated by A83-01 (Fig. 3c,d). We were not able to examine the effect TGFβ1 treatment on BEAS-2B cells deprived of serum as well as the effect of A83-01 on MCF-7 cells in the presence of TGFβ1 and absence of FBS due to substantial cell death (data not shown). Collectively, these findings suggested that S100A10 expression is regulated by TGFβ1 and is not necessarily linked to the epithelial or mesenchymal morphology of the cell.

### PI3kinase signaling represses S100A10 expression via FOXC2

The serum withdrawal experiment with the three cell lines also suggested the potential involvement of growth pathways in the regulation of S100A10 under EMT-inducing conditions. This is particularly relevant since TGFβ1, in addition to inducing EMT, inhibited cell growth (Supplemental Fig. 3a) concomitant with S100A10 upregulation. Growth factors are potent activators of receptor tyrosine kinases which trigger intracellular pro-growth signals^43^. In addition, the mechanism of action of the growth factor FGF is mediated through the activation of two pathways namely MAPK/MEK/Erk and PI3K/Akt/mTOR. Ramos *et al*. recently demonstrated that inhibition of both pathways prevented the restoration of E-cadherin expression in response to bhFGF in A549 cells treated with TGFβ1^41^. To examine the involvement of these pro-growth pathways during TGFβ1-indcued EMT, we treated A549 cells with the MEK inhibitor U0126 and the PI3K inhibitor LY294002 in the presence of TGFβ1. Inhibition of MEK did not affect S100A10 expression in the presence/absence of TGFβ1 (Supplemental Fig. 3b,c). In contrast, PI3K inhibition increased S100A10 protein expression, an effect that was then exacerbated by TGFβ1 in A549 (Fig. 3e) and MCF-7 (Fig. 3f) cells in a dose-dependent manner (supplemental Fig. 3d). S100A10 upregulation was also achieved when cells were treated with the mTOR inhibitor rapamycin (Supplemental Fig. 3e). In some cell models such as the murine breast epithelial cell line NMuMG, TGFβ1 promotes EMT via potent activation of PI3K through Akt phosphorylation followed by activation of mTORC1 (mammalian TOR complex 1) and mTORC2 in the NMuMG^44,45^. The latter represents a classic EMT model where the inhibition of PI3K hinders TGFβ1-induced EMT^45^. Inhibition of PI3K or inactivation of Akt abrogated TGFβ1-mediated activation of mTOR. The treatment of NMuMG cells with TGFβ1 resulted in downregulation of S100A10 expression consistent with the PI3K dependency of EMT in this cell line. These results implicate the PI3K/mTOR axis in regulating S100A10 in addition to the TGFβ1/SMAD4 pathway.

**Figure 4:**
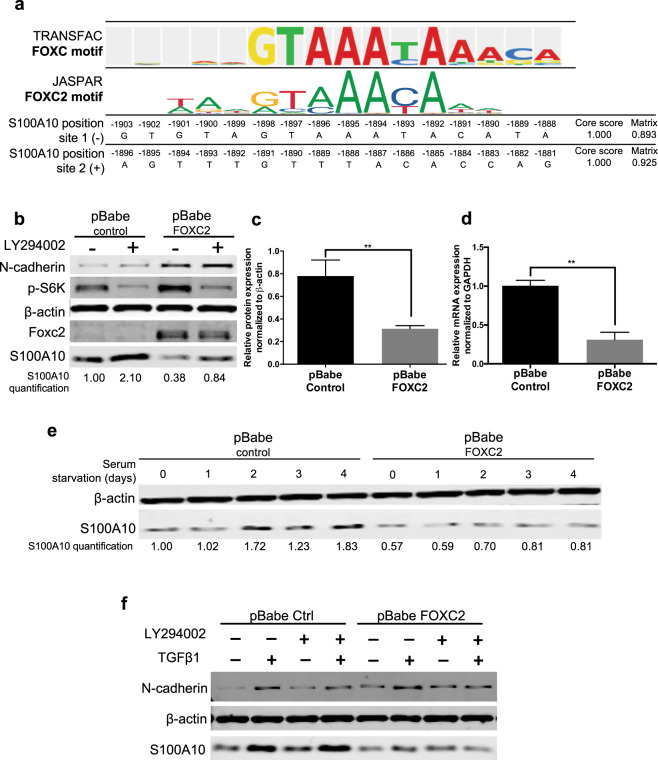
PI3K suppresses S100A10 expression through a FOXC2-mediated mechanism. TRANSFAC analysis of the only FOXC site in the S100A10 promoter region (2000bp upstream and 1000 bp downstream of transcription start site). The FOXC2 motif was extracted from Jaspar to compare similarity with TRANSFAC prediction (**a**). Western blot analysis (**b**), S100A10 protein quantification (**c**) and S100A10 mRNA quantification (**d**) of A549 cells transduced with pBabe-control and pBabe-FOXC2 vectors. Western blot of pBabe control and pBabe FOXC2 A549 cells treated with TGFβ1 in the presence/absence of serum for four consecutive days (**e**) or LY294002 (72 hours) (**f**).

A recent CHIP-chip analysis of the transcription factor *FOXC2* DNA binding sites revealed that the *S100A10* gene promoter contains the highly-conserved *de novo* motif (GCCAACAAAAACA, chr1: 150,219,126–150,220,276)^46^. TRANSFAC binding site motifs were selected and enriched based on their positional weight matrix. Three FOX binding sites were detected of which two were FOXC sites (site 1: GTGTAGTAAATACATA (−), site 2: AGTTTGTTTACACCAG (+)). We also confirmed the FOXC2 matrix profile using Jaspar (Fig. 4a). The expression of FOXC2 has been shown to be modulated by insulin via the PI3K signaling pathway^47,48^. In A549 cells, inhibition of PI3K by LY294002 reduced FOXC2 expression^49^ (Fig. 4b). Ectopic expression of FOXC2 also increased phosphorylation of S6K (Fig. 4b) and partially rescued the growth of LY294002-treated cells with no effect on TGFβ1-treated A549 cells (Supplemental Fig. 4c). To verify whether FOXC2 regulates S100A10 expression via PI3K signaling, A549 cells were transfected with the pBabe-FOXC2 construct. Ectopic FOXC2 expression caused a dramatic downregulation of S100A10 protein (Fig. 4b,c) and mRNA levels (Fig. 4d). In contrast, knockdown of FOXC2 resulted in an increase in S100A10 expression (Supplemental Fig. 4d).

Since PI3K inhibition increases S100A10 expression, we examined whether the downstream inhibitory effect of FOXC2 on S100A10 can abrogate the upregulation. Indeed, ectopic expression of FOXC2 sustained the downregulation of S100A10 in the presence of LY294002 (Fig. 4b). Similarly, serum withdrawal, that normally upregulated S100A10, failed to do so when FOXC2 was expressed (Fig. 4e). FOXC2 also maintained S100A10 downregulation in the presence of TGFβ1 (Fig. 4f). Collectively, these results indicate that S100A10 expression is positively modulated by canonical SMAD-dependent TGFβ1 signaling and negatively by growth factor signaling pathways such as PI3K/mTOR via a FOXC2-dependent mechanism.

### S100A10 serves as a plasminogen receptor at the surface of A549 cells

**Figure 5:**
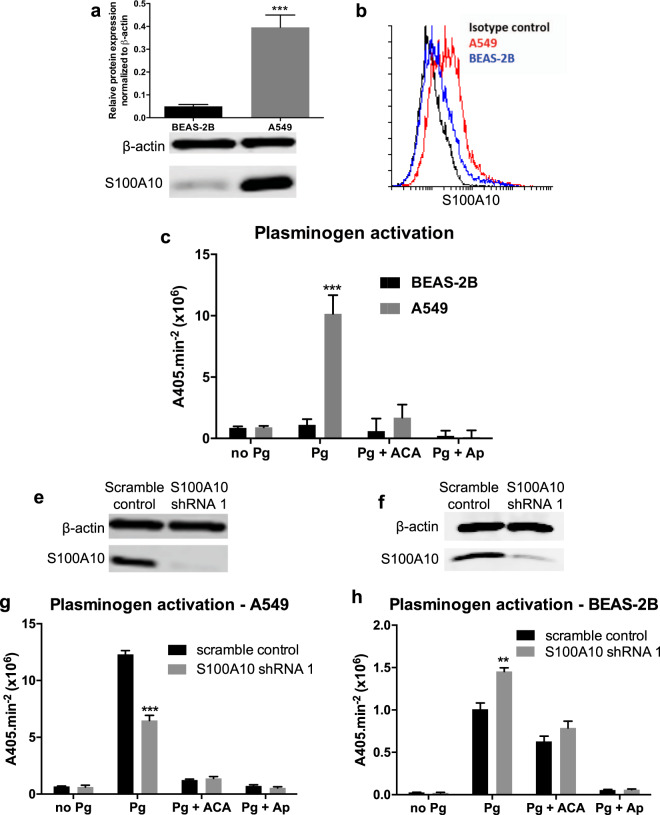
Surface S100A10 levels modulate plasminogen activation. Western blot analysis (**a**) and quantification (**b**) of total S100A10 protein and (**c**) flow cytometry of surface S100A10 levels in A549 and BEAS-2B cells. (**d**) Plasminogen activation assay of A549 and BEAS-2B cells in the presence of the lysine mimetic ε-aminocaproic acid (ACA) and protease inhibitor aprotinin (Ap). Western blot analysis of total S100A10 protein in scramble and S100A10-depleted (S100A10 shRNA 1) A549 (**e**) and BEAS-2B (**f**) cells. Plasminogen activation assay of A549 (**g**) and BEAS-2B (**h**) transfected with scramble control and S100A10 shRNA 1.

Since S100A10 is a well-established plasminogen receptor^50^, we examined how surface levels of S100A10 modulate plasminogen activation. We first compared total and surface S100A10 levels between the bronchial epithelial cell line BEAS-2B and the adenocarcinoma cell line A549 cells using flow cytometry. Both total (Fig. 5a) and surface (Fig. 5b) S100A10 protein expression were significantly higher in A549 cells compared to BEAS-2B cells. The difference in S100A10 expression was concomitant with differences in the ability of these cells to activate plasminogen (Fig. 5c). In addition, we depleted S100A10 in both cell lines using a stable shRNA knockdown (Fig. 5e,f). The depletion reduced plasminogen activation by 45% at the cell surface of A549 cells compared to the scramble control (Fig. 5g). ACA treatment (see methods) completely abolished plasminogen activation indicating that plasminogen binding to plasminogen receptors is the rate limiting step under these conditions. The remaining 55% was likely contributed by other plasminogen receptors (Fig. 5g). To avoid any potential compensation mechanisms upon stable shRNA knockdown, transient siRNA S100A10 knockdown (Supplemental Fig. 5a) resulted in a similar reduction in plasminogen activation (Supplemental Fig. 5b). In contrast, S100A10 depletion using shRNA (Fig. 5f) or siRNA (Supplemental Fig. 5c) in BEAS-2B cells did not decrease plasminogen activation compared to the scramble control which could be partly attributed to the low surface S100A10 levels. Additionally, ACA treatment did not completely abolish activation suggesting a low expression of plasminogen receptors with C-terminal lysines at the cell surface. These findings suggest that surface S100A10 serves as a plasminogen receptor and its expression is crucial for maintaining the activation of plasminogen. However, how the aforementioned changes in S100A10 induced by TGFβ1 or serum-withdrawal can affect plasminogen activation were yet to be addressed.

### Mesenchymal cells downregulate S100A10 surface expression and demonstrate a low capacity to activate plasminogen

**Figure 6:**
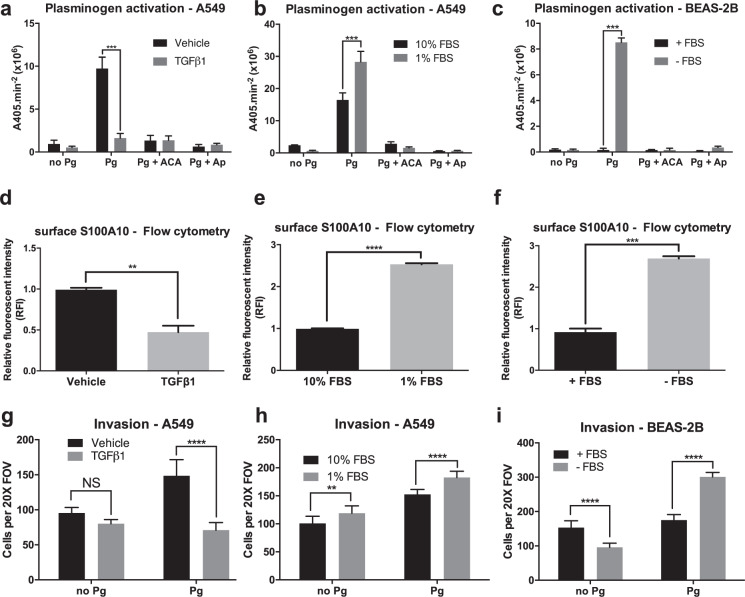
Plasminogen activation is partially dictated by the surface localization of plasminogen receptor S100A10 and not by the mesenchymal/epithelial state of A549 and BEAS-2B cells. Plasminogen activation assay and invasion assay of TGFβ1-treated A549 cells (**a** and **g** respectively), serum-deprived A549 cells (**b**,**h** respectively) and serum-deprived BEAS-2B cells (**c** and I respectively). Flow cytometry analysis/quantification of surface S100A10 expression and upon TGFβ1 treatment in A549 cells (**d**), FBS withdrawal in A549 cells (**e**) and FBS withdrawal in BEAS-2B cells (**f**).

We first examined how induction of epithelial- and mesenchymal-like morphology affects plasminogen activation especially in terms of S100A10 surface expression. To our surprise, TGFβ1 treatment abolished the ability of A549 cells to activate plasminogen at the cell surface (Fig. 6a). In addition and despite the upregulation of total S100A10 levels by TGFβ1 in A549 cells (Fig. 2b,c), surface S100A10 levels were also significantly reduced as shown using flow cytometry (Fig. 6d) and surface biotinylation (Supplemental Fig. 6a,b). Predictably, S100A10 knockdown did not affect plasminogen activation by TGFβ1-treated cells (Supplemental Fig. 6c). Serum withdrawal from A549 cells, which produced an epithelial-like morphology, significantly increased plasminogen activation (Fig. 6b) (Supplemental Fig. 6d). In addition, serum withdrawal from BEAS-2B cells restored plasminogen activation at the cell surface (Fig. 6c). Serum withdrawal from both A549 and BEAS-2B cells was concomitant increase in surface expression of S100A10 (Fig. 6e,f). Collectively, these results suggested that these mesenchymal cells possess a low capacity to activate plasminogen, which is partly attributable to low surface S100A10 levels.

Since enhanced plasminogen activation has been positively linked to increased cancer cell invasion^51^, we assessed how the low plasminogen capacity of mesenchymal-like cells alters their *in vitro* invasiveness. A549 cells were treated with TGFβ1 for 4 days after which they were seeded into Boyden invasion chambers. In the absence of plasminogen, TGFβ1-treated A549 cells did not have an effect on the ability of these cells to penetrate the underlying matrigel. The addition of 0.5 μM plasminogen increased invasiveness of vehicle-treated A549 cells. Interestingly, TGFβ1-treated cells were not responsive to the exogenous plasminogen-dependent increase in invasion (Fig. 6g). This is consistent with the low plasminogen activation capacity of TGFβ1-treated cells. Similarly, upon serum withdrawal, epithelial-like A549 and BEAS-2B cells were more responsive to increase in plasminogen-dependent invasion (Fig. 6h,i). Together, these findings indicated that epithelial-like cells have an enhanced ability to activate plasminogen at the cell surface and are more proficient at plasminogen-dependent invasion (Supplemental Fig. 7).

### S100A10 and uPAR-mediated plasminogen activation is potentially masked by dramatic PAI-1 upregulation

**Figure 7:**
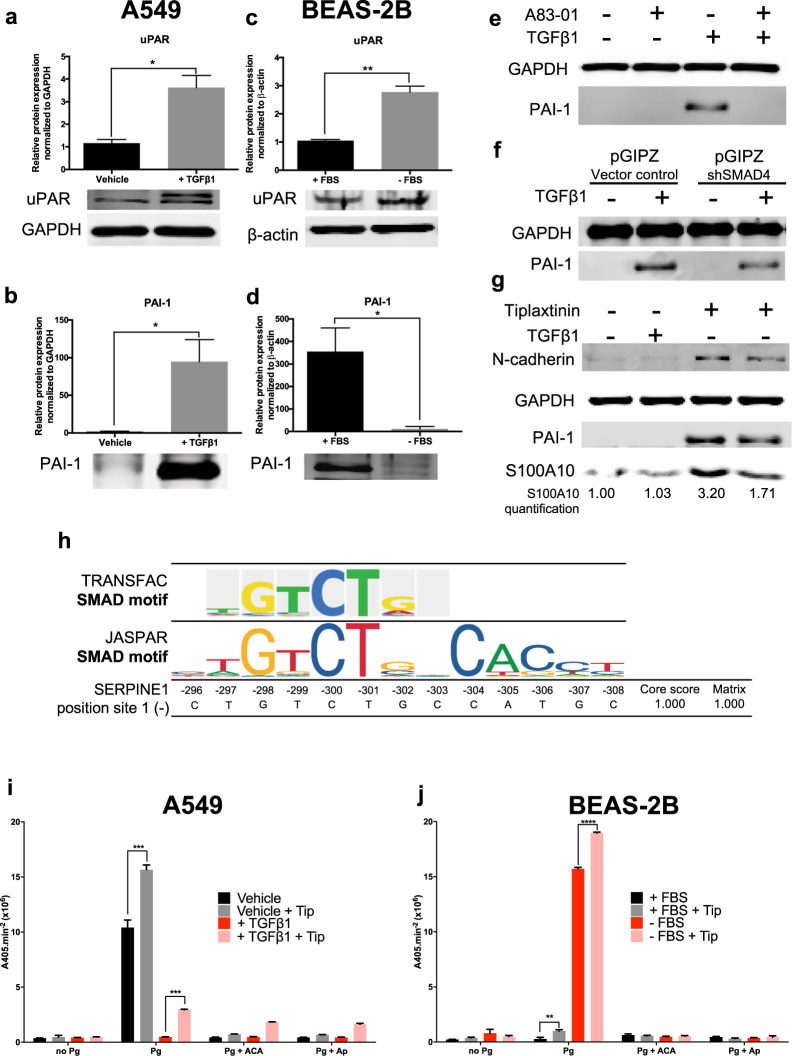
S100A10, PAI-1 and uPAR are differentially regulated in epithelial-like and mesenchymal-like A549 and BEAS-2B cells. Western blot analysis and quantification of uPAR and PAI-1 in vehicle-treated and TGFβ1-treated A549 cells (**a**,**b**) and in BEAS-2B +/− FBS cells (**c**,**d**). Western blot analysis of PAI-1 in A549 cells either treated with A83-01 (**e**) or depleted from SMAD4 (**f**) +/−TGFβ1. Western blot analysis (**g**) and plasminogen activation assay of A549 cells (**h**) and BEAS-2B cells (**i**) treated with PAI-1 inhibitor tiplaxtinin (10 μM) in +/−TGFβ1.

The low rate of plasminogen activation in TGFβ1-treated and serum-supplemented cells was unlikely to be entirely attributable to the decrease in S100A10 surface levels. In an attempt to understand the mechanism of surface plasminogen activation in these cells, we assessed the contribution of other components of the PA system. Specifically, we focused on PAI-1 which binds to and inactivates uPA and on uPAR which binds uPA and facilitates uPA-dependent plasminogen activation. Both genes encoding PAI-1 and uPAR were upregulated by TGFβ1 by 835-fold and 9.64-fold respectively (Fig. 2a) (Supplemental Table 1). We first confirmed uPAR (Fig. 7a) and PAI-1 (Fig. 7b) upregulation in TGFβ1-treated A549 cells. In contrast, uPAR was upregulated (Fig. 7c) while PAI-1 was downregulated (Fig. 7d) in BEAS-2B cells upon withdrawal of serum, consistent with the dramatic increase in plasminogen activation. The elevated expression of PAI-1 by TGFβ1 indicated that both S100A10 and PAI-1 might be regulated by the similar mechanisms. Indeed, PAI-1 upregulation by TGFβ1 was inhibited by A83-01 (Fig. 7e) and abrogated by SMAD4 knockdown (Fig. 7f). TRANSFAC analysis revealed a SMAD binding site on the (−) DNA strand around 300 bp upstream of the transcription start site. The site also matched the SMAD2:3:4 binding site as determined by JASPAR (Fig. 7h) (Supplemental Fig. 4b). In addition, FOXC2 expression in A549 cells induced PAI-1 expression (Supplemental Fig. 4e). Interestingly, our TRANSFAC analysis showed no putative binding sites for FOX or FOXC transcription factors in the assessed region (3000 bp; 2000 upstream and 1000 downstream of transcription site) of PAI-1 (Supplemental Fig. 4b).

Since PAI-1 is a potent inhibitor of plasminogen activation, we assessed whether its inhibition by tiplaxtinin could rescue plasminogen activation in TGFβ1-treated A549 and serum-supplemented BEAS-2B cells. Only partial inhibition (45%) of PAI-1 was achieved with minimal cellular toxicity (Fig. 7g). Nonetheless, tiplaxtinin increased plasminogen activation in vehicle-treated A549 cells and partially restored activation in TGFβ1-treated cells (Fig. 7i). Both serum-supplemented and serum-starved BEAS-2B cells treated with tiplaxtinin showed a similar but less dramatic increase in plasminogen activation (Fig. 7j). These results indicate that high PAI-1 expression in mesenchymal cells greatly contributed to quenching global plasminogen activation at the cell surface. PAI-1 low expression in epithelial cells enables these cells to have enhanced plasminogen activation capabilities driven by other plasminogen modulators such as S100A10 and uPAR.

## Discussion

The coupled cellular processes of EMT and MET play well-established roles in organ formation during embryogenesis and tissue regeneration during wound healing^1^. However, a plethora of studies over the last two decades have suggested that aberrant activation of EMT/MET can promote tumor cell invasion and malignant tumor progression^6^. Although contentious, it is currently believed that EMT is involved in the early invasive escape of cancer cells from the primary tumor site whilst MET is associated with metastatic site seeding and repopulation^52,53^. In a parallel manner, the plasminogen activation system has been known to play a key role in tumorigenesis and metastasis^51,54^. It was however unknown how the plasminogen activation system and specifically the plasminogen receptors are regulated during EMT/MET. Here we demonstrated for the first time that in cells undergoing TGFβ1-induced EMT, a select group of plasminogen activation proteins are differentially induced. Although cells possess multiple plasminogen receptors^22^ that contribute to plasminogen activation, We here reported that S100A10 was the only plasminogen receptor regulated by TGFβ1-induced EMT, suggesting that S100A10 is a key regulator of the plasminogen activation system during TGFβ1-induced EMT., The depletion of S100A10 in A549 cells resulted in marked decrease in plasminogen activation, which is likely justified by an adequate level of S100A10 expression at the cell surface. In addition, we previously identified S100A10 as a key plasminogen receptor that empowers stromal cells and many cancer cells with the ability to promote plasminogen activation during malignant progression of cancer cells^27,29,55^. Furthermore, a recent mass spectrometric comparison of epithelial-like and mesenchymal-like ARCaP prostate cancer cells revealed that S100A10 was one of 76 proteins that were significantly increased in the mesenchymal-like cells^56^. Similarly, Keshamouni *et al*. used iTRAP labeling and mass spectrometry to demonstrate that S100A10 was one of 27 proteins that were upregulated in A549 cells during TGFβ1-induced EMT^57^. A recent study also showed that the levels of miR-590 are decreased in an EMT model *in vitro* and *in vivo*. S100A10 is a known target of miR-590 which inhibits S100A10 expression^58^. The dependency of S100A10 upregulation on the expression of wild-type SMAD4 was manifested in the absence of a response in the pancreatic cell line BxPC-3 which harbors a *SMAD4* homozygous deep deletions. Ali *et al*. revealed that reactivation of mutant SMAD4 in HCT116 colorectal cancer cells upregulated a series of S100 proteins including S100A2, S100A4, S100A10 and S100A11^59^. Our findings consolidate these observations by directly demonstrating the robust increase in S100A10 protein expression during TGFβ-induced EMT.

In the context of EMT, the impact of S100A10 expression on plasminogen activation was not linked to the epithelial or mesenchymal state of the cell but rather to the surface expression of S100A10. Despite the upregulation of total S100A10 expression by TGFβ1, plasminogen activation, cellular invasiveness as well as surface S100A10 expression were dramatically reduced. In addition, the plasminogen activation inhibitor PAI-1 was upregulated in mesenchymal cells which markedly reduced their plasminogen activation potential. Both PAI-1 and S100A10 were modulated through canonical TGFβ1/SMAD signaling as well as through PI3K signaling via the transcription factor FOXC2. Importantly, mesenchymal cells were less likely to invade through Matrigel in response to plasminogen compared to epithelial cells. The lack of increase in invasion in response to exogenous plasminogen is most likely attributed to the low capability of mesenchymal cells to activate plasminogen primarily due to high PAI-1 levels. Interestingly, TGFβ1 did not increase invasion even in the absence of plasminogen, which at first glance, seems contradictory to previous studies which clearly demonstrate that TGFβ1 increases invasion of A549 cells (Supplemental Fig. 7)^60,61^. However, this could partly be explained by methodological differences where we pre-treated cells for four days with TGFβ1 at which point they became mesenchymal and were then seeded into the trans-well Boyden chambers and counted after 24 hours. The comparison examined the invasive properties of epithelial cells compared to their mesenchymal derivatives. Meanwhile, previous studies addressing the impact of TGFβ1 on invasion were performed where A549 cells were seeded directly into the trans-well chambers along with TGFβ1 and invading cells were counted after 24 hours. The later methodology addresses how TGFβ1 alters invasion within 24 hours of exposure without sufficient induction of EMT. Our results challenge the existing models that indicate that EMT increases *in vitro* cell invasiveness. The question of how *in vitro* EMT-controlled invasiveness translates into *in vivo* cellular dissemination and metastasis remains a contentious topic. The fact the mesenchymal cells are more likely to escape primary tumors does not necessitate that these same cells will give rise to metastatic growth. Indeed, the recent advent of mouse models that allow EMT lineage tracing of tumor cells has offered new insights into the role of EMT in metastasis *in vivo*. A 2015 report by Fischer *et al*. demonstrated that epithelial and not mesenchymal cancer cells were largely responsible for lung metastases formation in breast cancer. Instead, EMT contributed to resistance to the chemotherapeutic agent cyclophosphamide^62^. Similarly, Zheng *et al*. reported that EMT induced by Twist and Snail was dispensable for metastasis in a mouse model of pancreatic cancer^63^. A 2014 report also demonstrated that expression of E-cadherin, whose loss is considered a hallmark of EMT, increased invasiveness of cancer cells *in vitro*^64^. These studies challenge the concept that mesenchymal cells in primary tumors are solely responsible for the dissemination process that initiates metastasis. EMT-dependency and metastasis have become matters of contention primarily due to their context-dependency.

In addition to canonical SMAD-mediated signaling, TGFβ1 activates non-canonical pathways such as PI3K, MAPK and Rho-like GTPases. The pro-growth Akt/PI3K has been previously demonstrated to either negatively or positively complement the biological and morphological changes associated with EMT^65^. For instance, PI3K and Akt inhibited apoptosis induced by TGFβ1 via the interaction of Akt with SMAD3 preventing SMAD3 phosphorylation in Hep3B and HEK293T cells^66,67^. In cell models including those described in this study, the cross-talk between TGFβ1 and PI3K signaling pathways produced antagonist effects on S100A10 expression. The inhibition of PI3K/mTOR or the withdrawal of serum in the presence of TGFβ1 increased S100A10 expression partly due to direct SMAD signaling as well as alleviating the inhibition of SMADs by PI3K (Fig. 3e,f). Evidently, the activation of PI3K by FGF-1 prevented the upregulation of S100A10 by TGFβ1 (Fig. 2h, Supplemental Fig. 2e). Whether the dependency of TGFβ1-induced EMT on PI3K activation is a universal mechanism remains elusive and is highly context-specific^68^. Some earlier evidence suggested that the PI3K-dependency is present in systems where TGFβ1-mediated signaling was not reliant on SMADs to downregulate E-cadherin and upregulate N-cadherin as seen in NMuMG cells^69^. Not surprisingly, treating NMuMG cells with TGFβ1 resulted in downregulation of S100A10 expression consistent with the PI3K dependency in this cell line (Supplemental Fig. 3f,g). Notably, the modulation of S100A10 expression was not linked to N-cadherin expression indicating that S100A10 is a TGFβ1- and PI3K-regulated gene and not an “EMT gene”. This became more evident where serum withdrawal, known to diminish PI3K signaling, induced an epithelial-like morphology and increased S100A10 expression (Supplemental Fig. 2c).

**Figure 8:**
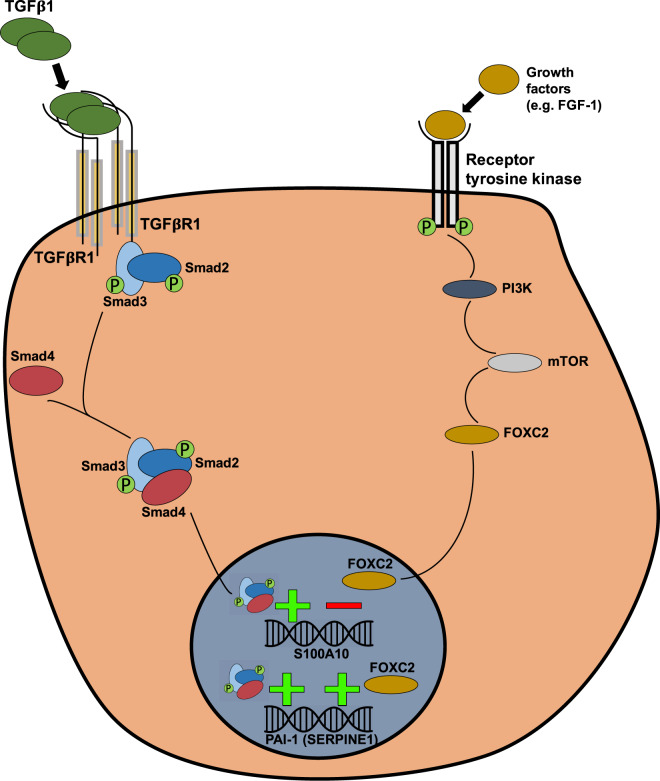
S100A10 and PAI-1 are regulated by Smad4-dependent TGFβ1-mediated signaling and FOXC2-mediated PI3K signaling. The model proposes that the treatment of epithelial cells with TGFβ1 increases S100A10 and PAI-1 mRNA and protein levels through canonical Smad-dependent TGFβ1 signaling. S100A10 and PAI-1 are also affected by the pro-growth PI3K pathway. Serum starvation, PI3K inhibition or mTOR inhibition upregulate S100A10 expression suggesting an inhibitory effect through this pathway. The transcription factor FOXC2, which is downstream of PI3K, mediates the repression of S100A10 expression and induction of PAI-1.

Serum-supplemented BEAS-2B or A549 cells possessed a reduced capability to activate plasminogen, which could be increased when an epithelial phenotype was induced (Fig. 6a–c). Serum-starved cells may represent a more epithelial-like state as evident by increased E-cadherin expression (Fig. 1b,d,f). Dong Su *et al*. demonstrated that the epithelial-like morphology of A549 caused by serum withdrawal was mediated via c-src activation and subsequent upregulation of E-cadherin^33^. Our findings indicated the first association between the epithelial and mesenchymal state of cells and their differential capacity to activate plasminogen (Fig. 8). Importantly, the lack of plasminogen activation in mesenchymal A549 and BEAS-2B cells (Fig. 6d,f respectively) could not solely be explained by the low surface levels of S100A10 since S100A10 depletion only yielded a 45% decrease in plasminogen activation by A549 cells (Fig. 5g). This suggested the involvement of other components of the PA system such as uPAR and PAI-1. PAI-1 was likely the major contributor to quenching plasminogen activation (Fig. 8). since its inhibition partially restored plasminogen activation in A549 (Fig. 7i) and BEAS-2B (Fig. 7j) cells. In contrast, uPAR and S100A10 upregulation coupled with PAI-1 downregulation contributed to the drastic increase in plasminogen activation by BEAS-2B cells upon serum withdrawal. Interestingly, both uPAR signaling and PAI-1 expression have been shown to be required for activation of EMT in breast cancer cells^70^ and fibroblasts^71^ respectively. It is plausible that TGFβ1-mediated activation of EMT was further compounded by the concurrent activation of PAI-1 and uPAR. In that context, S100A10 expression was downregulated when PAI-1 was inhibited (Figs 7g and 8).

The suppression of S100A10 by PI3K was likely mediated through a FOXC2-dependent mechanism (Figs 4b and 8). The transcription factor FOXC2 belongs to the forkhead-box family of transcription factors and is required for the maturation of the primary lymphatic plexus into collecting lymphatic vessels during embryonic development^46^. FOXC2 has also been implicated in oncogenic progression^72^ and in promoting EMT via downregulating E-cadherin expression in A549 cells^49^ and increasing N-cadherin (Fig. 4b). Since FOXC1 have similar functions to FOXC2, we examined whether TGFβ1-induced EMT also induces FOXC1. TGFβ1 treatment in A549, MCF-7 and BEAS-2B cells did not induce FOXC1 expression (Supplemental Fig. 6e). Importantly, Yu *et al*. also demonstrated that FOXC2 expression was driven by PI3K signaling and not by canonical TGFβ1 signaling^49^ (Fig. 4b). In fact, FOXC2 overexpression in A549 cells treated with the anti-proliferative inhibitor LY294002 partially restored their growth capability (Supplemental Fig. 4b) confirming that FOXC2 is indeed downstream of PI3K in A549 cells. Interestingly, FOXC2 was reported to be linked to higher plasma levels of PAI-1 and TGFβ1 during intravascular thrombosis^73–75^. Our TRANSFAC analysis failed to identify SMAD binding sites in the PAI-1 gene promoter. Howver, a 2006 study by Fujita *et al*. demonstrated that FOXC2 does bind upstream of the PAI-1 gene in response to TGFβ1 (through SMADs) or to insulin (through PI3K) in bovine and human endothelial cells^47,48^ (Supplemental Fig. 4e). It is plausible that the site identified by Fujita *et al*. is novel and has not been added to the TRANSFAC repertoire.

## Methods

### Contact for reagent and resource sharing

Further information and requests for resources and reagents should be directed to and will be fulfilled by the lead contact, David Waisman (david.waisman@dal.ca). There are no restrictions for use of materials and reagents.

### Method details

#### Cell lines

All cell lines were purchased from the American Type Culture Collection (ATCC) (except HMLE and BxPC-3) and all tested negative for mycoplasma. A549 (CCL-185, male), NMuMG (CRL-1636, female) and MCF-7 (HTB-22, female) cells were supplemented with Dulbecco’s Modified Eagle’s Media (DMEM) with 10% fetal bovine serum (Hyclone). BEAS-2B (CRL-9609, male) were supplemented with LHC-8 media (Thermo-fisher scientific) with and without FBS (Hyclone, Canada, characterized). Panc 10.05 (CRL-2547, male) and BxPC-3 cells (CRL-1687, female), a generous gift from Dr. David Hoskin, Dalhousie University, were supplemented with Roswell Park Memorial Institute (RPMI) with 10% fetal bovine serum. HMLE (human mammary epithelial cell line, female) cells were a generous gift from Dr. Robert Weinberg (Whitehead Institute for Biomedical Research, Cambridge, Massachusetts) and were cultured in a 1:1 ratio of DMEM F12 1:1 and mammary epithelial cell growth medium (MEGM, Lonza) supplemented with 13 µg/mL bovine pituitary extract, 20 µg/mL human epidermal growth factor,10 µg/mL insulin, 1 µg/mL gentamicin/amphotericin and 2 µg/mL hydrocortisone (Lonza Clonetics) and 10% FBS. All cells were cultured in the presence of 1% pencillin/streptomycin (Hyclone) and were maintained at 37 °C with 5% CO_2_.

#### Antibodies

β-actin (Sigma Aldrich mouse monoclonal anti-β-actin, A2228, 1:2000), 42 kDa. N-cadherin (BD Biosciences mouse monoclonal anti-N-cadherin, 610921, 1:2000) 120 kDa. E-cadherin (BD Biosciences mouse monoclonal anti-E-cadherin, 610181, 1:2000) 120 kDa. Vimentin (Sigma-Aldrich goat polyclonal anti-Vimentin, V4630, 1:1000) 58 kDa. S100A10(BD Biosciences mouse monoclonal anti-S100A10, 610070, 1:2000) 11 kDa. Annexin A2 (BD Biosciences mouse monoclonal anti-Annexin II, 610069, 1:2000) 36 kDa. GAPDH (Biochain mouse monoclonal anti-GAPDH, Y3322, 1:2000) 36 kDa. p-S6K (Cell signaling rabbit monoclonal anti-pS6K, 9205 S, 1:1000) 70 kDa. FOXC2 (Bethyl laboratories rabbit polyclonal anti-FOXC2, A302-383A, 1:1000) 65–70 kDa. PAI-1 (Cell signaling rabbit monoclonal anti-PAI-1 D9C4, 11907, 1:2000) 48 kDa. uPAR (Santa Cruz rabbit polyclonal anti-uPA H149, sc-10815, 1:300) 55 kDa. p-ERK (Erk1/2) (Cell signaling rabbit polyclonal anti-Erk1/2 (Thr202/Tyr204), 9101, 1:1000) 42, 44 kDa.

#### Transfections and plasmids

The S100A10 shRNA1 knockdown construct was designed by cloning the following dsRNA oligo 5′-GAT CCC CGT GGG CTT CCA GAG CTT CTT TCA AGA GAA GAA GCT CTG GAA GCC CAC TTT TTA-3′ and 5′-AGC TTA AAA AGT GGG CTT CCA GAG CTT CTT CTC TTG AAA GAA GCT CTG GAA GCC CAC GGG-3′ into the pSUPER-retro-puro vector plasmid (OligoEngine). The non-silencing siRNA (4390843) and S100A10 siRNA (s12429) were purchased from the Ambion Silencer Select pre-designed and validated siRNA library (ThermoFisher Scientific). The plasmid vectors pBabe-puro-Control (#1764) and pBabe-puro-FOXC2 (#15535) were obtained from the plasmid depository Addgene. The pGIPZ SMAD4 and FOXC2 constructs were obtained from EGAD (enhanced Gene Analysis and Discovery) core facility at Dalhousie University. All transfected cell lines were selected and maintained in 1 µg/ml puromycin.

#### Reagents and inhibitors

All reagents were optimized for ideal dosage and time courses to minimize cellular toxicity while maximizing response of proteins of interest. TGFβ1 (Peprotech, 20 ng/ml unless indicated), LY294002 (Santa Cruz Biotechnology, 50 µM), Rapamycin (Tocris, 10 µM), A83-01(Tocris, 25 µM), Tiplaxtinin (Tocris, 10 µM), bhFGF-1 (basic human fibroblast growth factor-1 constituted in 100 ug/ml heparin) (R&D systems, 0 to 200 ng/ml), heparin sodium salt (Tocris, 100 ug/ml).

#### Western blot

Cells were washed with PBS and lysed in lysis buffer (1% NP-40, 150 mM NaCl, 20 mM Tris, pH 7.0, 1 mM EDTA and 1 mM EGTA) containing 2X Halt protease and phosphatase inhibitors (ThermoScientific). Samples were subject to SDS-polyacrylamide gel electrophoresis then transferred onto a nitrocellulose membrane. Membranes were incubated with primary antibodies overnight at 4 °C or one hour at room temperature. Li-COR secondary antibodies used to visualize bands using a LI-COR Odyssey imaging scanner.

#### Quantitative RT-PCR

RNA was extracted using TRIzol as per standard procedure (Qiagen). 2 µg of RNA was used for the synthesis of cDNA using Superscript II (Invitrogen). *S100A10* (S100A10) gene expression was amplified using gene-specific primers on the CFX96™ platform. *S100A10* primers were as follows: Forward primer GAGTGCTCATGGAACGGGAG and reverse GTGGGGCAGATTCCTCAAGT (IDT). Relative mRNA expression was calculated using the Livak and Schmittgen’s 2^−∆∆CT^ method and normalized to GAPDH as a reference gene^76^.

#### Plasminogen activation assay

Cells were seeded overnight into 96-well plates at 1 × 10^5^ cells/well. Cells were then washed Dulbecco’s PBS (Hyclone), incubated with 0.5 µM plasminogen for 10 minutes and then incubated with 0.5 mM S2251 (chromogenic plasmin substrate, Chromogenix, Diapharma Group). The rate of plasminogen activation was quantified based on the absorbance at 405 nm every 4 minutes for 4 hours using the Spectra M3 plate reader (Molecular Devices). A405nm was subtracted from A600 to account for turbidity. ε-aminocaproic acid (ACA), a lysine analog, serves as a plasminogen activation inhibitor via inhibiting plasminogen binding to its receptors. Aprotinin (Ap) is a serine protease inhibitor and serves as internal negative control to confirm that the S2251 cleavage was mediated by a serine protease i.e. plasmin.

#### Invasion assay

1 × 10^5^ cells were seeded in serum-free media into the upper chamber of a trans-well Boyden chamber with 8 µm pores (BD Biosciences). The bottom chamber contained 10% FBS as a chemoattractant. 0.5 µM plasminogen was added to the top chambers 5 hours after seeding. After 72 hours, transversed cells were stained with 0.2% crystal violet and counted (Five fields of view per membrane at 20X magnification).

#### Flow cytometry

Cells were washed with PBS, gently lifted with a cell lifter and then blocked with 2% FBS in PBS. Cells were then incubated with primary antibodies at room temperature for 30 minutes, washed 3 times with PBS then incubated with FITC- or PE-conjugated secondary antibodies for 30 min at room temperature. Cells were then washed with PBS and analyzed on a BD FACSCalibur flow cytometer.

#### Surface biotinylation

Cells were seeded into 15-cm cell culture plates until 90% confluency. Cells were then washed twice with ice-cold PBS and incubated with 1 mg/ml Sulfo-NHS-SS-Biotin (Pierce, Thermo Scientific) for 30 min at 4 °C. The reaction was quenched with 100 µM glycine in PBS, then washed twice with ice-cold PBS. Cells were then lysed in RIPA lysis buffer. 500 µg of protein lysates were incubated with 100 µl of Dynabeads M-280 streptavidin (Invitrogen) for 2 hours at 4 °C with rotation. Biotinylated proteins were separated from unlabeled proteins using a magnet with five washes of the lysis buffer. Biotinylated proteins were then suspended in protein sample buffer, boiled at 95 °C for 10 min and subjected to gel electrophoresis.

#### H&E staining

Cells were seeded on Poly-L-Lysine slides then fixed and permeabilized using 1:1 ratio of methanol and acetone. Fixed cells were then stained with hematoxylin, washed with PBS, then stained with eosin. Glass slides were then mounted for bright-field microscope imaging (Zeiss).

### Quantification and statistical analysis

All experiments were performed in triplicates in three independent experiments. All statistical analysis was performed using GraphPad Prism 5 software. Statistical significance based on differences in the mean (+/−standard deviation) was determined using the unpaired two-tailed student t-test or one-way ANOVA accordingly. A significance threshold of *P*-value < 0.05 was applied. *P* < 0.05 *P < 0.01 **P < 0.001 ***P < 0.0001 **** For western blot quantification, data are represented as mean ± SD.

#### Quantification of western blot band intensities

Band intensity was measured using the Odyssey Li-COR software V3.0. The intensity was then subtracted from background intensity (above or below band). Relative band intensities per lane were determined for each protein and normalized to intensities of GAPDH or β-actin bands. Noteworthy, a consistent upregulation of β-actin was observed in A549 cells in response to TGFβ1 treatment (Fig. 1b). Protein expression was therefore normalized relative to GAPDH under conditions where A549 cells were treated with TGFβ1. All gels were cropped to improve clarity. Molecular weights of proteins are listed under the antibodies section.

#### Quantification of flow cytometry relative fluorescent intensities (RFIs)

Surface expression was quantified using the flowing software 2 based on relative fluorescence intensities (RFIs). RFI was calculated by subtracting the mean fluorescence intensity of samples incubated with the anti-S100A10 antibody from that of samples incubated with IgG1 isotype control.

#### Gene array analysis and normalization

Gene expression of 130 components of the plasminogen activation system was examined in a published expression array (GSE17708, Affymetrix HG_U133_plus_2) of A549 cells treated with 5 ng/ml TGFβ1 for multiple time points. Gene expression at the 72 hr time point of TGFβ1-treated cells was compared to the untreated control cells^36^. The expression of 9 reference genes (*TBP*, *ACTB*, *RPL32*, *GAPDH*, *EF1A*, *CYCC*, *HPRT1*, *B2M* and *ALB*) was examined for data normalization. The gene *EF1A* (elongation factor 1α) was the most reliable gene (with almost no divergence between untreated and treated samples) (Supplemental Fig. 2a). Gene expression values were converted to anti-ln values (2^expression value) after which they were subtracted from the anti-ln expression values of *EF1A*. Normalized expression values are listed in Supplemental Table 1 and are represented as a volcano plot in Fig. 2a.

## Electronic supplementary material

Supplemental file

## References

[1] Kalluri R, Weinberg RA (2009). The basics of epithelial-mesenchymal transition. Journal of Clinical Investigation.

[2] Kalluri R (2009). EMT: When epithelial cells decide to become mesenchymal-like cells. Journal of Clinical Investigation.

[3] Derynck R, Zhang YE (2003). Smad-dependent and Smad-independent pathways in TGF-β family signalling. Nature.

[4] Deckers M (2006). The tumor suppressor Smad4 is required for transforming growth factor β-induced epithelial to mesenchymal transition and bone metastasis of breast cancer cells. Cancer Res..

[5] Nieto MA (2013). Epithelial Plasticity: A Common Theme in Embryonic and Cancer Cells. Science (80-.).

[6] Tiwari N, Gheldof A, Tatari M, Christofori G (2012). EMT as the ultimate survival mechanism of cancer cells. Seminars in Cancer Biology.

[7] Puisieux A, Brabletz T, Caramel J (2014). Oncogenic roles of EMT-inducing transcription factors. Nat. Cell Biol..

[8] Ye X, Weinberg RA (2015). Epithelial-Mesenchymal Plasticity: A Central Regulator of Cancer Progression. Trends in Cell Biology.

[9] Mani SA (2008). The Epithelial-Mesenchymal Transition Generates Cells with Properties of Stem Cells. Cell.

[10] Schmidt A, Echtermeyer F, Alozie A, Brands K, Buddecke E (2005). Plasmin- and thrombin-accelerated shedding of syndecan-4 ectodomain generates cleavage sites at Lys114-Arg115 and Lys 129-Val130 bonds. J. Biol. Chem..

[11] Tjwa M (2009). Fibrinolysis-independent role of plasmin and its activators in the haematopoietic recovery after myeloablation. J. Cell. Mol. Med..

[12] Liotta LA (1981). Effect of Plasminogen Activator (Urokinase), Piasmin, and Thrombin on Glycoprotein and Collagenous Components of Basement Membrane. Cancer Res.

[13] Gechtman, Z., Sharma, R., Kreizman, T., Fridkin, M. & Shaltiel, S. Synthetic peptides derived from the sequence around the plasmin cleavage site in vitronectin. *FEBS Letters* **315** (1993). 10.1016/0014-5793(93)81181-x 7678553

[14] Presta M (2005). Fibroblast growth factor/fibroblast growth factor receptor system in angiogenesis. Cytokine and Growth Factor Reviews.

[15] Matsuoka H, Sisson TH, Nishiuma T, Simon RH (2006). Plasminogen-mediated activation and release of hepatocyte growth factor from extracellular matrix. Am. J. Respir. Cell Mol. Biol..

[16] McColl BK (2003). Plasmin activates the lymphangiogenic growth factors VEGF-C and VEGF-D. J. Exp. Med..

[17] Deryugina, E. I. & Quigley, J. P. Cell surface remodeling by plasmin: A new function for an old enzyme. *Journal of Biomedicine and Biotechnology* **2012** (2012). 10.1155/2012/564259 PMC3477900 23097597

[18] Majumdar M (2004). Plasmin-induced migration requires signaling through protease-activated receptor 1 and integrin α9β1. J. Biol. Chem..

[19] Rijken, D. C. & Sakharov, D. V. Basic principles in thrombolysis: Regulatory role of plasminogen. In *Thrombosis Research* **103** (2001). 10.1016/s0049-3848(01)00296-1 11567668

[20] Ellis, V. The Plasminogen Activation System in Normal Tissue Remodeling. In *Matrix Proteases in Health and Disease*, 10.1002/9783527649327.ch2 25–55 (2012).

[21] Danø K (2005). Plasminogen activation and cancer. Thromb. Haemost..

[22] Miles, L. A., Plow, E. F., Waisman, D. M. & Parmer, R. J. Plasminogen receptors. *Journal of Biomedicine and Biotechnology* **2012** (2012). 10.1155/2012/130735 PMC3511834 23226936

[23] Mutch NJ, Thomas L, Moore NR, Lisiak KM, Booth NA (2007). TAFIa, PAI-1 and α2-antiplasmin: Complementary roles in regulating lysis of thrombi and plasma clots. J. Thromb. Haemost.

[24] Bianchi E (1995). Immunohistochemical localization of the plasminogen activator inhibitor-1 in breast cancer. Int J Cancer.

[25] Nielsen BS (2001). Urokinase plasminogen activator is localized in stromal cells in ductal breast cancer. Lab. Invest..

[26] Dass K, Ahmad A, Azmi AS, Sarkar SH, Sarkar FH (2008). Evolving role of uPA/uPAR system in human cancers. Cancer Treatment Reviews.

[27] Phipps KD, Surette AP, O’Connell PA, Waisman DM (2011). Plasminogen receptor S100A10 is essential for the migration of tumor-promoting macrophages into tumor sites. Cancer Res..

[28] Bydoun M, Waisman DM (2014). On the contribution of S100A10 and annexin A2 to plasminogen activation and oncogenesis: an enduring ambiguity. Future Oncol.

[29] Madureira, P. A., O’Connell, P. A., Surette, A. P., Miller, V. A. & Waisman, D. M. The biochemistry and regulation of S100A10: A multifunctional plasminogen receptor involved in oncogenesis. *Journal of Biomedicine and Biotechnology* **2012** (2012). 10.1155/2012/353687 PMC3479961 23118506

[30] Doerner, A. M. & Zuraw, B. L. TGF-β1 induced epithelial to mesenchymal transition (EMT) in human bronchial epithelial cells is enhanced by IL-1β but not abrogated by corticosteroids. *Respir. Res*, 10.1186/1465-9921-10-100 (2009). 10.1186/1465-9921-10-100 PMC2774671 19857272

[31] Zhao F, Klimecki WT (2015). Culture conditions profoundly impact phenotype in BEAS-2B, a human pulmonary epithelial model. J. Appl. Toxicol..

[32] Kawata M (2012). TGF-β-induced epithelial-mesenchymal transition of A549 lung adenocarcinoma cells is enhanced by pro-inflammatory cytokines derived from RAW 264.7 macrophage cells. J. Biochem..

[33] Dong S (2014). Serum starvation regulates E-cadherin upregulation via activation of c-Src in non-small-cell lung cancer A549 cells. Am. J. Physiol. Cell Physiol..

[34] Lv Z-D (2013). Transforming growth factor-β 1 enhances the invasiveness of breast cancer cells by inducing a Smad2-dependent epithelial-to-mesenchymal transition. Oncol. Rep.

[35] Kondo Y (2012). Induction of epithelial-mesenchymal transition by flagellin in cultured lung epithelial cells. AJP Lung Cell. Mol. Physiol.

[36] Sartor MA (2009). ConceptGen: A gene set enrichment and gene set relation mapping tool. Bioinformatics.

[37] Miles LA, Plow EF (1985). Binding and activation of plasminogen on the platelet surface. J. Biol. Chem..

[38] Tojo M (2005). The ALK-5 inhibitor A-83-01 inhibits Smad signaling and epithelial-to-mesenchymal transition by transforming growth factor-β. Cancer Sci..

[39] Le Gendre O (2013). Suppression of AKT Phosphorylation Restores Rapamycin-Based Synthetic Lethality in SMAD4-Defective Pancreatic Cancer Cells. Mol. Cancer Res..

[40] Jinnin M (2005). Characterization of SIS3, a Novel Specific Inhibitor of Smad3, and Its Effect on Transforming Growth Factor-beta1-Induced Extracellular Matrix Expression. Mol. Pharmacol..

[41] Ramos C, Becerril C (2010). FGF-1 reverts epithelial-mesenchymal transition induced by TGF-β1 through MAPK/ERK kinase pathway. Am. J. ….

[42] Wingender, E., Dietze, P., Karas, H. & Knüppel, R. TRANSFAC: A database on transcription factors and their DNA binding sites. *Nucleic Acids Research*, 10.1093/nar/24.1.238 (1996). 10.1093/nar/24.1.238 PMC145586 8594589

[43] Slomovitz BM, Coleman RL (2012). The PI3K/AKT/mTOR pathway as a therapeutic target in endometrial cancer. Clinical Cancer Research.

[44] Lamouille S, Derynck R (2007). Cell size and invasion in TGF-??-induced epithelial to mesenchymal transition is regulated by activation of the mTOR pathway. J. Cell Biol..

[45] Lamouille S, Connolly E, Smyth JW, Akhurst RJ, Derynck R (2012). TGF–induced activation of mTOR complex 2 drives epithelial-mesenchymal transition and cell invasion. J. Cell Sci..

[46] Norrmén C (2009). FOXC2 controls formation and maturation of lymphatic collecting vessels through cooperation with NFATc1. J. Cell Biol.

[47] Fujita H (2006). Foxc2 is a common mediator of insulin and transforming growth factor beta signaling to regulate plasminogen activator inhibitor type I gene expression. Circ. Res..

[48] Madamanchi NR, Runge MS (2006). Five easy pieces: The obesity paradigm. Circulation Research.

[49] Yu Y-H (2013). MiR-520h-mediated FOXC2 regulation is critical for inhibition of lung cancer progression by resveratrol. Oncogene.

[50] Kwon M, MacLeod TJ, Zhang Y, Waisman DM (2005). S100A10, annexin A2, and annexin a2 heterotetramer as candidate plasminogen receptors. Front. Biosci..

[51] Kwaan HC, McMahon B (2009). The role of plasminogen-plasmin system in cancer. Cancer Treatment and Research.

[52] Jolly, M. K., Ware, K. E., Gilja, S., Somarelli, J. A. & Levine, H. EMT and MET: necessary or permissive for metastasis? *Molecular Oncology*, 10.1002/1878-0261.12083 (2017). 10.1002/1878-0261.12083 PMC5496498 28548345

[53] Smith, B. & Bhowmick, N. Role of EMT in Metastasis and Therapy Resistance. *J. Clin. Med*., 10.3390/jcm5020017 (2016). 10.3390/jcm5020017 PMC4773773 26828526

[54] Andreasen Pa, Egelund R, Petersen HH (2000). The plasminogen activation system in tumor growth, invasion, and metastasis. Cell. Mol. Life Sci..

[55] O’Connell PA, Surette AP, Liwski RS, Svenningsson P, Waisman DM (2010). S100A10 regulates plasminogen-dependent macrophage invasion. Blood.

[56] Zhang, S. *et al*. EPLIN downregulation promotes epithelial-mesenchymal transition in prostate cancer cells and correlates with clinical lymph node metastasis. *Oncogene*, 10.1038/onc.2011.199 (2011). 10.1038/onc.2011.199 PMC3165108 21625216

[57] Keshamouni, V. G. *et al*. Differential protein expression profiling by iTRAQ-2DLC-MS/MS of lung cancer cells undergoing epithelial-mesenchymal transition reveals a migratory/invasive phenotype. *J. Proteome Res*., 10.1021/pr050455t (2006). 10.1021/pr050455t 16674103

[58] Shan X (2013). MiR-590-5p inhibits growth of HepG2 cells via decrease of S100A10 expression and inhibition of the wnt pathway. Int. J. Mol. Sci..

[59] Ali NA, McKay MJ, Molloy MP (2010). Proteomics of Smad4 regulated transforming growth factor-beta signalling in colon cancer cells. Mol. Biosyst..

[60] Xu, C.-C. *et al*. Effects of TGF-β signaling blockade on human A549 lung adenocarcinoma cell lines. *Mol. Med. Rep*., 10.3892/mmr.2011.530 (2011). 10.3892/mmr.2011.530 21725601

[61] Xu, G. *et al*. Cisplatin sensitivity is enhanced in non-small cell lung cancer cells by regulating epithelial-mesenchymal transition through inhibition of eukaryotic translation initiation factor 5A2. *BMC Pulm. Med*., 10.1186/1471-2466-14-174 (2014). 10.1186/1471-2466-14-174 PMC4232729 25380840

[62] Fischer KR (2015). Epithelial-to-mesenchymal transition is not required for lung metastasis but contributes to chemoresistance. Nature.

[63] Zheng X (2015). Epithelial-to-mesenchymal transition is dispensable for metastasis but induces chemoresistance in pancreatic cancer. Nature.

[64] Liu X, Huang H, Remmers N, Hollingsworth MA (2014). Loss of E-cadherin and epithelial to mesenchymal transition is not required for cell motility in tissues or for metastasis. Tissue Barriers.

[65] Bakin AV, Tomlinson AK, Bhowmick NA, Moses HL, Arteaga CL (2000). Phosphatidylinositol 3-kinase function is required for transforming growth factor beta-mediated epithelial to mesenchymal transition and cell migration. J. Biol. Chem..

[66] Conery AR (2004). Akt interacts directly with Smad3 to regulate the sensitivity to TGF-beta induced apoptosis. Nat. Cell Biol..

[67] Remy I, Montmarquette A, Michnick SW (2004). PKB/Akt modulates TGF-β signalling through a direct interaction with Smad3. Nat. Cell Biol..

[68] Larue L, Bellacosa A (2005). Epithelial–mesenchymal transition in development and cancer: role of phosphatidylinositol 3′ kinase/AKT pathways. Oncogene.

[69] Bhowmick NA (2001). Transforming growth factor-beta1 mediates epithelial to mesenchymal transdifferentiation through a RhoA-dependent mechanism. Mol. Biol. Cell.

[70] Jo M (2009). Reversibility of epithelial-mesenchymal transition (EMT) induced in breast cancer cells by activation of urokinase receptor-dependent cell signaling. J. Biol. Chem..

[71] Omori K (2016). Inhibition of Plasminogen Activator Inhibitor-1 Attenuates Transforming Growth Factor-β-Dependent Epithelial Mesenchymal Transition and Differentiation of Fibroblasts to Myofibroblasts. PLoS One.

[72] Cui YM (2015). FOXC2 promotes colorectal cancer metastasis by directly targeting MET. Oncogene.

[73] Cederberg A (2001). FOXC2 is a winged helix gene that counteracts obesity, hypertriglyceridemia, and diet-induced insulin resistance. Cell.

[74] Brunen D (2013). TGF-β: An emerging player in drug resistance. Cell Cycle.

[75] Oshimori N, Oristian D, Fuchs E (2015). TGF-β Promotes Heterogeneity and Drug Resistance in Squamous Cell Carcinoma. Cell.

[76] Livak KJ, Schmittgen TD (2001). Analysis of relative gene expression data using real-time quantitative PCR and the 2(−Delta Delta C(T)) Method. Methods.

