# Author Correction: A novel mechanism of plasminogen activation in epithelial and mesenchymal cells

**DOI:** 10.1038/s41598-020-68535-9

**Published:** 2020-07-13

**Authors:** Moamen Bydoun, Andra Sterea, Ian C. G. Weaver, Alamelu G. Bharadwaj, David M. Waisman

**Affiliations:** 1 Department of Pathology, Halifax, Nova Scotia Canada; 2 Department of Physiology and Biophysics, Halifax, Nova Scotia Canada; 3 Department of Biochemistry and Molecular Biology, Halifax, Nova Scotia Canada; 4 Department of Psychology and Neuroscience, Halifax, Nova Scotia Canada; 5 Department of Psychiatry, Halifax, Nova Scotia Canada; 6 Brain Repair Centre, Dalhousie University, Halifax, Nova Scotia Canada

Correction to: *Scientific Reports* 10.1038/s41598-018-32433-y, published online 20 September 2018

The original version of this Article contained a typographical error in the spelling of the author Alamelu G. Bharadwaj, which was incorrectly given as Alamelu Dharini Bharadwaj. This has now been corrected in the PDF and HTML versions of the Article, and in the accompanying Supplementary Information file.

The Author Contributions section now reads:

“Conceptualization M.B. and D.M.W. Methodology M.B. Formal Analysis M.B. and A.S. Investigation M.B., A.S., I.C.G.W. and A.G.B. Resources D.M.W. and I.C.G.W. Visualization M.B., A.S., I.C.G.W. writing – Original draft M.B. Writing – Review & Editing M.B., A.S. and D.M.W. supervision D.M.W. Funding acquisition D.M.W.”

