# Supplementary material for: A novel mechanism of plasminogen activation in epithelial and mesenchymal cells
Source: Sci Rep. 2018 Sep 20;8:14091. doi: 10.1038/s41598-018-32433-y (PMC6148250; doi:10.1038/s41598-018-32433-y)
Supplement: Supplementary file 1 — Supplemental file [file 41598_2018_32433_MOESM1_ESM.pdf]

A novel mechanism of plasminogen activation in epithelial and mesenchymal cells

Moamen Bydoun[1], PhD., Andra Sterea[2], BSc. Ian C.G. Weaver[1,4,5,6], Alamelu G. Bharadwaj[3], PhD. and David M. Waisman[1,3] PhD.

[1]Department of Pathology, [2]Department of Physiology and Biophysics, [3]Department of Biochemistry and Molecular Biology, [4]Department of Psychology and Neuroscience, [5]Department of Psychiatry, [6]Brain Repair Centre, Dalhousie University, Halifax, Nova Scotia, Canada.

Address: Charles Tupper Medical Building, 5850 College Street, 11L1, B3H4R2, Halifax, Nova Scotia, Canada

Correspondence

David.Waisman@dal.ca

PO Box 15000
902-494-1803 (office)
902-494-2766 (lab)
902-494-1355 (fax)

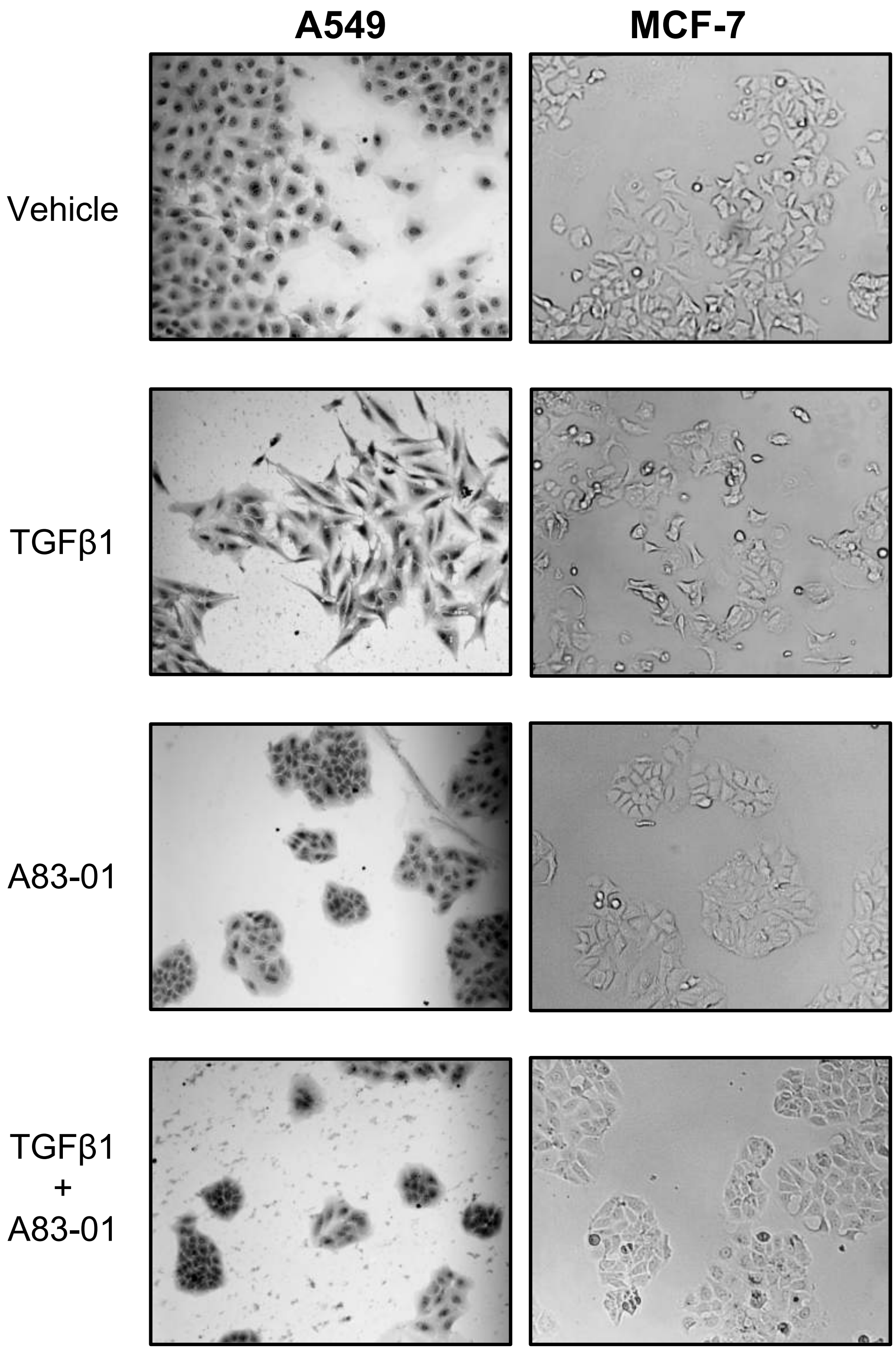

Supplemental figure 1. TGFβ1 treatment of A549 and MCF-7 cells induces a mesenchymal-like morphology and can be reversed by TGFβR1 inhibition. Related to figure 1. TGFβ1 induces a morphological change in both A549 and MCF-7 cells to become fibroblast-like mesenchymal cells compared to vehicle-treated cells. This change can be inhibited by the TGFβR1 inhibitor (A83-01) in vehicle-treated and TGFβ1-treated A549 and MCF-7 cells. A83-01 generated a epithelial- like phenotype that appears to be more epithelial than the vehicle-treated cells.

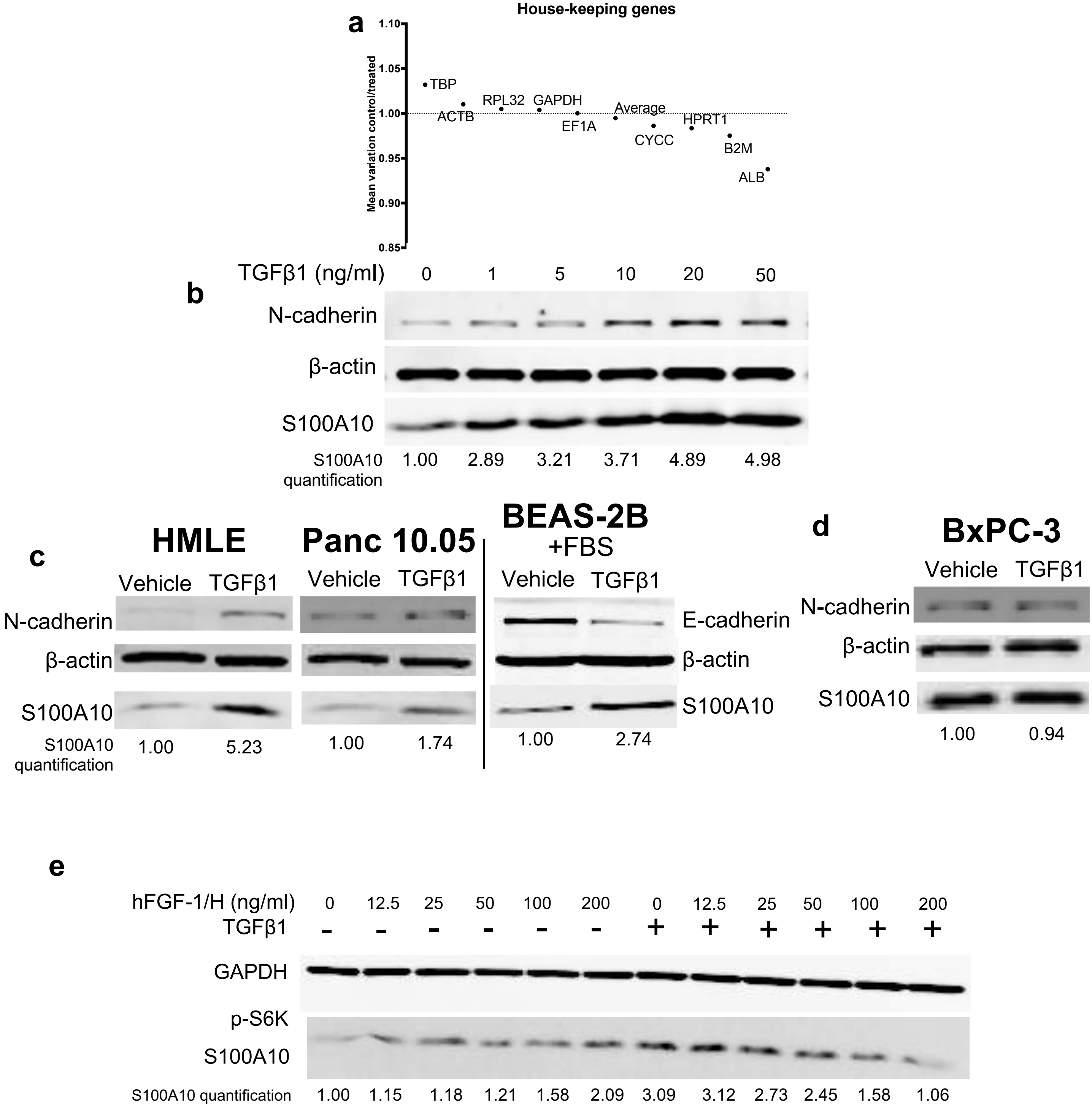

Supplemental figure 2. TGFβ1 treatment upregulates S100A10 protein expression in multiple cancer cell lines. Related to figure 2. (a) Identification of the most reliable house-keeping gene based on change of expression between untreated and treated samples. A value of "1" indicates no change in expression between untreated and treated samples. (b) Western blot analysis of S100A10 in A549 cells treated with a increasing concentrations of TGFβ1 (0, 1, 5, 10, 20 and 50 ng/ml). Western blot analysis of S100A10 in (c) HMLE , Panc 10.05, MCF-7 and BEAS-2B cells and (d) BxPC-3 treated with 20 ng/ml TGFβ1 for 8, 3, 4, 4 and 4 consecutive days respectively. (e) Western blot analysis and quantification of protein lysates from vehicle-treated and TGFβ1-treated BEAS-2B cells in the presence of ascending concentrations of 0 to 200 ng/ml of bhFGF-1/H after 72 hours.

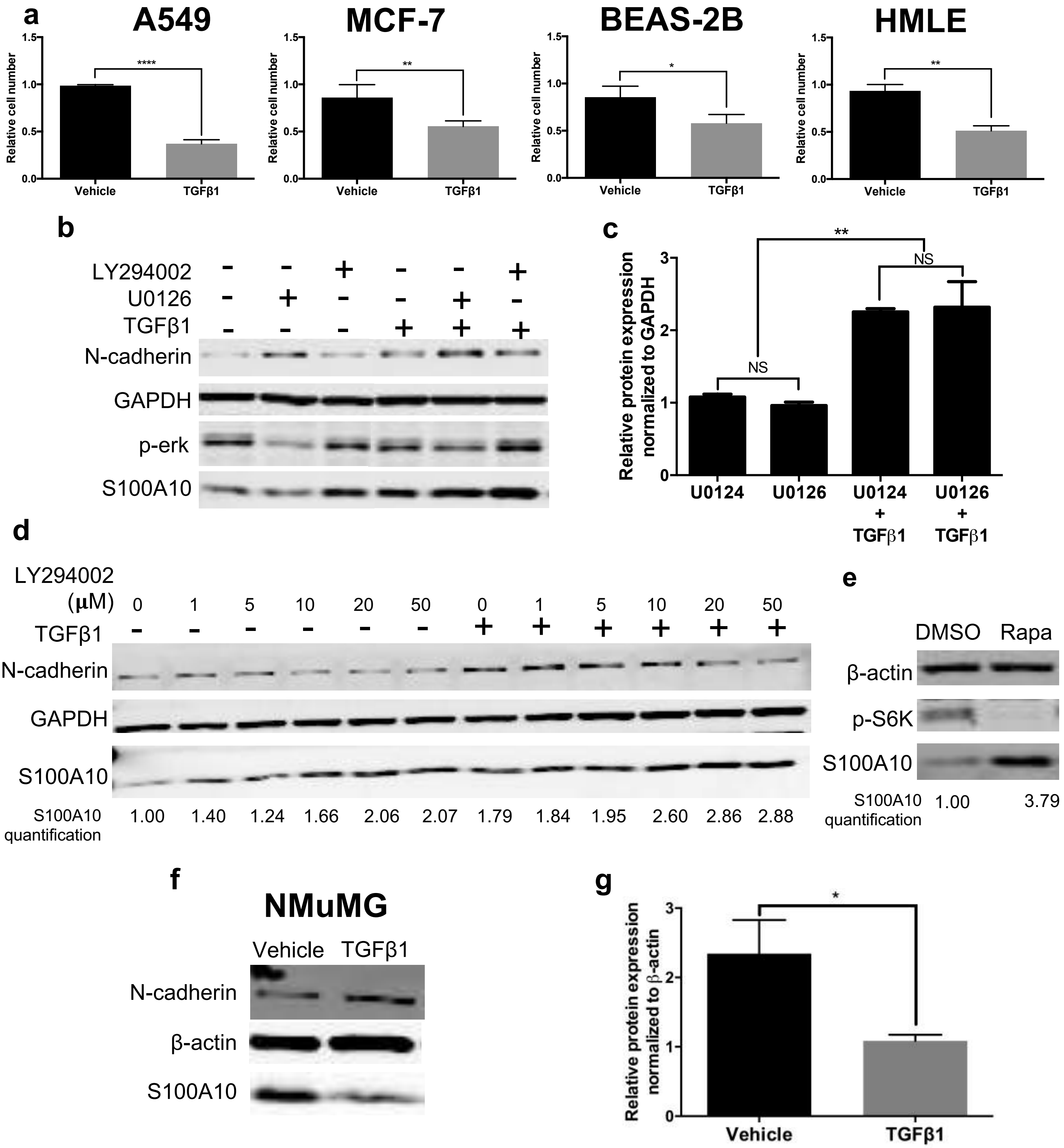

Supplemental figure 3. PI3K signaling suppresses S100A10 expression. Related to figure 3. (a) A549, MCF-7, BEAS-2B and HMLE cells were counted after 4 days (8 days for HMLE cells) of vehicle or TGFβ1 treatment using the Trypan blue dye. (b) Western blot analysis and S100A10 protein quantification (c) in A549 cells treated with the MEK inhibitor U0126 and PI3K inhibitor LY294002 in the presence or absence of TGFβ1. (d) Western blot analysis of S100A10 in A549 cells under treatment with increasing doses of LY294002 (0, 1, 5, 10, 20 and 50 **μ**M) in the presence or absence of TGFβ1. (e) Western blot analysis of S100A10 in A549 cells treated with rapamycin (10 **μ**M) for 48 hours. Western blot analysis (f) and quantification (g) of S100A10 in NMuMG cells treated with 20ng/ml TGFβ1 for three consecutive days.

a

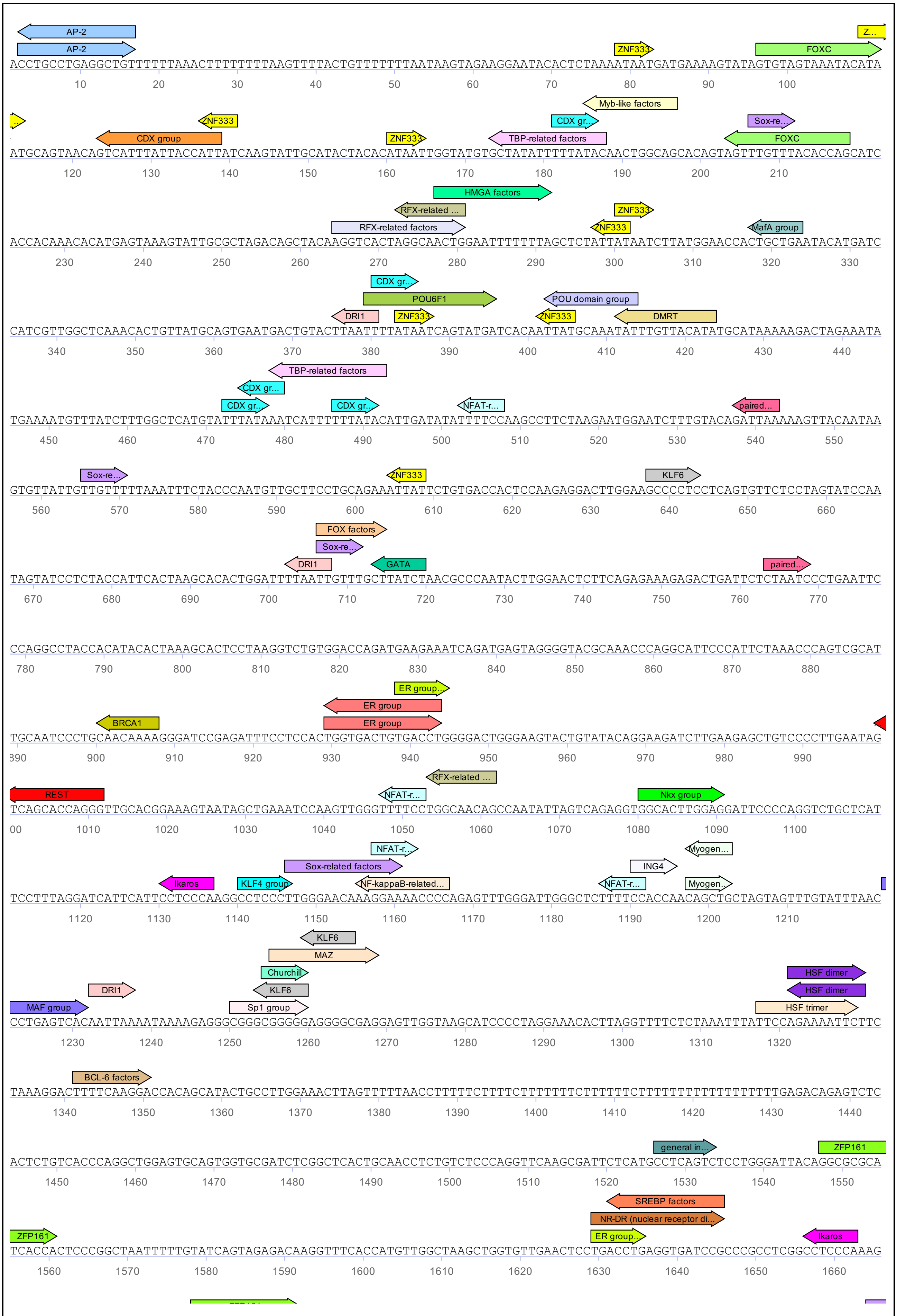

AP-2
AP-2
ZNF333
FOXC
Z...
ACCTGCCTGAGGCTGTTTTTTAAACTTTTTTTTAAGTTTTACTGTTTTTTTAATAAGTAGAAGGAATACACTCTAAAATAATGATGAAAAGTATAGTGTAGTAAATACATA
Myb-like factors
CDX gr...
Sox-re...
ZNF333
CDX group
ZNF333
TBP-related factors
FOXC
ATGCAGTAACAGTCATTTATTACCATTATCAAGTATTGCATACTACACATAATTGGTATGTGCTATATTTTTATACAACTGGCAGCACAGTAGTTTGTTTACACCAGCATC
HMGA factors
RFX-related ...
ZNF333
RFX-related factors
ZNF333
MafA group
ACCACAAACACATGAGTAAAGTATTGCGCTAGACAGCTACAAGGTCACTAGGCAACTGGAATTTTTTTAGCTCTATTATAATCTTATGGAACCACTGCTGAATACATGATC
CDX gr...
POU6F1
POU domain group
DRI1
ZNF333
ZNF333
DMRT
CATCGTTGGCTCAAACACTGTTATGCAGTGAATGACTGTACTTAATTTTATAATCAGTATGATCACAATTATGCAAATATTTGTTACATATGCATAAAAAGACTAGAAATA
TBP-related factors
CDX gr...
CDX gr...
CDX gr...
NFAT-r...
paired...
TGAAAATGTTTATCTTTGGCTCATGTATTTATAAATCATTTTTTATACATTGATATATTTTCCAAGCCTTCTAAGAATGGAATCTTTGTACAGATTAAAAGTTACAATAA
Sox-re...
ZNF333
KLF6
GTGTTATTGTTGTTTTTAAATTTCTACCCAATGTTGCTTCCTGCAGAAATTATTCTGTGACCACTCCAAGAGGACTTGGAAGCCCCTCCTCAGTGTTCTCCTAGTATCCAA
FOX factors
Sox-re...
DRI1
GATA
paired...
TAGTATCCTCTACCATTCACTAAGCACACTGGATTTTAATTGTTTGCTTATCTAACGCCCAATACTTGGAACTCTTCAGAGAAAGAGACTGATTCTCTAATCCCTGAATTC
CCAGGCCTACCACATACACTAAAGCACTCCTAAGGTCTGTGGACCAGATGAAGAAATCAGATGAGTAGGGGTACGCAAACCCAGGCATTCCCATTCTAAACCCAGTCGCAT
ER group...
ER group
BRCA1
ER group
TGCAATCCCTGCAACAAAAGGGATCCGAGATTTCCTCCACTGGTGACTGTGACCTGGGGACTGGGAAGTACTGTATACAGGAAGATCTTGAAGAGCTGTCCCCTTGAATAG
RFX-related ...
REST
NFAT-r...
Nkx group
TCAGCACCAGGGTTGCACGGAAAGTAATAGCTGAAATCCAAGTTGGGTTTTCCTGGCAACAGCCAATATTAGTCAGAGGTGGCACTTGGAGGATTCCCCAGGTCTGCTCAT
NFAT-r...
Myogen...
Sox-related factors
ING4
Ikaros
KLF4 group
NF-kappaB-related...
NFAT-r...
Myogen...
TCCTTTAGGATCATTCATTCCTCCCAAGGCCTCCCTTGGGAACAAAGGAAAACCCCAGAGTTTGGGATTGGGCTCTTTTCCACCAACAGCTGCTAGTAGTTTGTATTTAAC
KLF6
MAZ
Churchill
HSF dimer
DRI1
KLF6
HSF dimer
MAF group
Sp1 group
HSF trimer
CCTGAGTCACAATTAAAATAAAAGAGGGCGGGCGGGGGAGGGGCGAGGAGTTGGTAAGCATCCCCTAGGAAACACTTAGGTTTCTCTAAATTTATTCCAGAAAATTCTTC
BCL-6 factors
TAAAGGACTTTTCAAGGACCACAGCATACTGCCTTGGAAACTTAGTTTTTAACCTTTTTCTTTTCTTTTTTTCTTTTTTCTTTTTTTTTTTTTTTTTTTGAGACAGAGTCTC
general in...
ZFP161
ACTCTGTCACCCAGGCTGGAGTGCAGTGGTGCGATCTCGGCTCACTGCAACCTCTGTCTCCCAGGTTCAAGCGATTCTCATGCCTCAGTCTCCTGGGATTACAGGCGCGCA
SREBP factors
NR-DR (nuclear receptor di...
ZFP161
ER group...
Ikaros
TCACCACTCCCGGCTAATTTTTGTATCAGTAGAGACAAGGTTTCACCATGTTGGCTAAGCTGGTGTTGAACTCCTGACCTGAGGTGATCCGCCCGCCTCGGCCTCCCAAAG

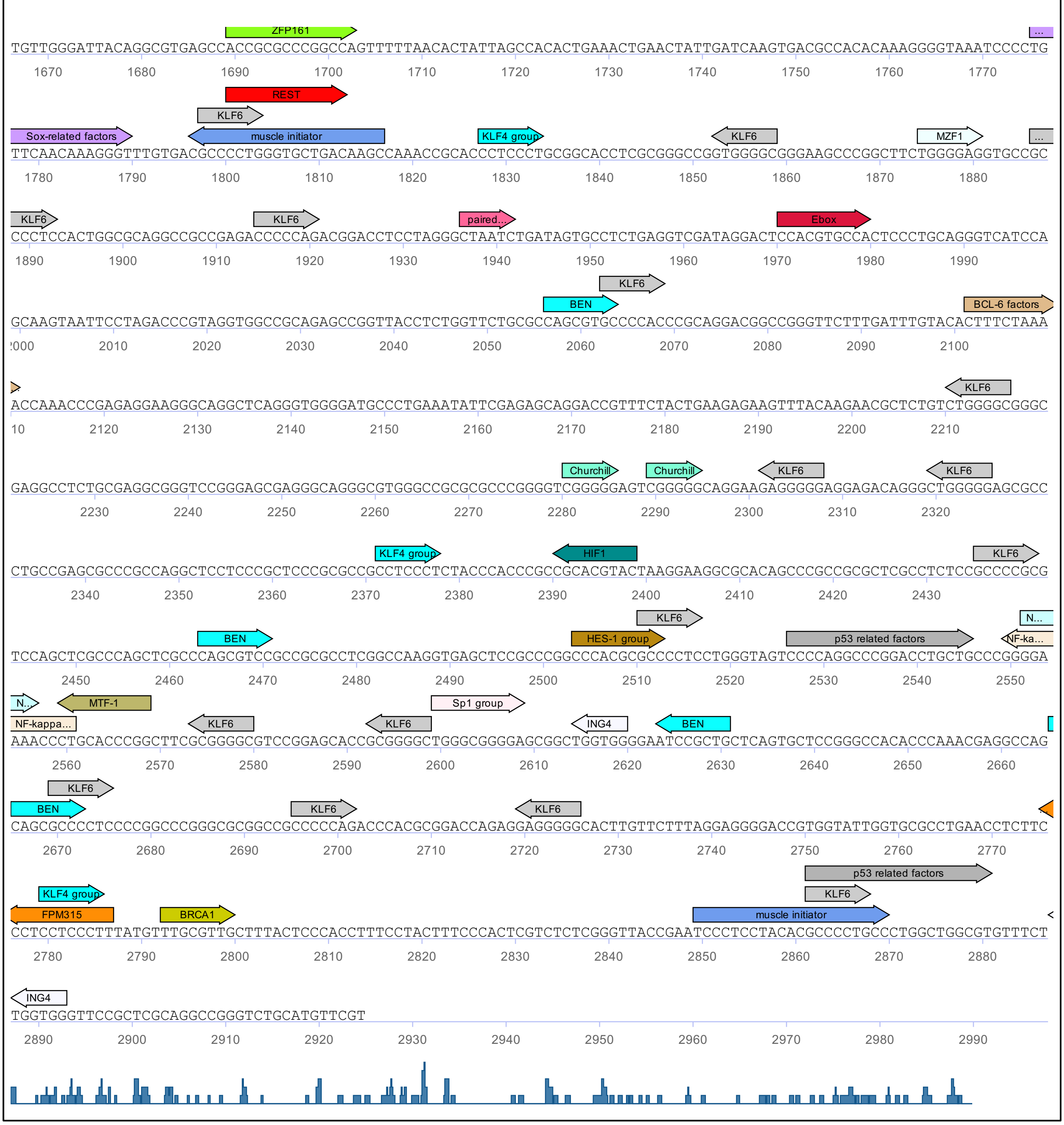

ZFP161
...
TGTTGGGATTACAGGCGTGAGCCACCGCGCCCGGCCAGTTTTTAACACTATTAGCCACACTGAAACTGAACTATTGATCAAGTGACGCCACACAAAGGGGTAAATCCCCTG
1670 1680 1690 1700 1710 1720 1730 1740 1750 1760 1770
REST
KLF6
Sox-related factors
muscle initiator
KLF4 group
KLF6
MZF1
...
TTCAACAAAGGGTTTGTGACGCCCCTGGGTGCTGACAAGCCAAACCGCACCCTCCCTGCGGCACCTCGCGGGCCGGTGGGGCGGGAAGCCCGGCTTCTGGGGAGGTGCCGC
1780 1790 1800 1810 1820 1830 1840 1850 1860 1870 1880
KLF6
KLF6
paired...
Ebox
CCCTCCACTGGCGCAGGCCGCCGAGACCCCCAGACGGACCTCCTAGGGCTAATCTGATAGTGCCTCTGAGGTCGATAGGACTCCACGTGCCACTCCCTGCAGGGTCATCCA
1890 1900 1910 1920 1930 1940 1950 1960 1970 1980 1990
KLF6
BEN
BCL-6 factors
GCAAGTAATTCCTAGACCCGTAGGTGGCCGCAGAGCCGGTTACCTCTGGTTCTGCGCCAGCGTGCCCCACCCGCAGGACGGCCGGGTTCTTTGATTTGTACACTTTCTAAA
2010 2020 2030 2040 2050 2060 2070 2080 2090 2100
KLF6
ACCAAACCCGAGAGGAAGGGCAGGCTCAGGGTGGGGATGCCCTGAAATATTCGAGAGCAGGACCGTTTCTACTGAAGAGAAGTTTACAAGAACGCTCTGTCTGGGGCGGGC
2120 2130 2140 2150 2160 2170 2180 2190 2200 2210
Churchill
Churchill
KLF6
KLF6
GAGGCCTCTGCGAGGCGGGTCCGGGAGCGAGGGCAGGGCGTGGGCCGCGCGCCCGGGGTCGGGGGAGTCGGGGGCAGGAAGAGGGGGAGGAGACAGGGCTGGGGGAGCGCC
2230 2240 2250 2260 2270 2280 2290 2300 2310 2320
KLF4 group
HIF1
KLF6
CTGCCGAGCGCCCGCCAGGCTCCTCCCGCTCCCGCGCCGCCTCCCTCTACCCACCCGCCGCACGTACTAAGGAAGGCGCACAGCCCGCCGCGCTCGCCTCTCCGCCCCGCG
2340 2350 2360 2370 2380 2390 2400 2410 2420 2430
KLF6
N...
BEN
HES-1 group
p53 related factors
NF-ka...
TCCAGCTCGCCCAGCTCGCCCAGCGTCCGCCGCGCCTCGGCCAAGGTGAGCTCCGCCCGGCCCACGCGCCCCTCCTGGGTAGTCCCCAGGCCCGGACCTGCTGCCCGGGGA
2450 2460 2470 2480 2490 2500 2510 2520 2530 2540 2550
N...
MTF-1
Sp1 group
NF-kappa...
KLF6
KLF6
ING4
BEN
AAACCCTGCACCCGGCTTCGCGGGGCGTCCGGAGCACCGCGGGGCTGGGCGGGGAGCGGCTGGTGGGGAATCCGCTGCTCAGTGCTCCGGGCCACACCCAAACGAGGCCAG
2560 2570 2580 2590 2600 2610 2620 2630 2640 2650 2660
KLF6
BEN
KLF6
KLF6
CAGCGCCCCTCCCCGGCCCGGGCGCGGCCGCCCCCAGACCCACGCGGACCAGAGGAGGGGGCACTTGTTCTTTAGGAGGGGACCGTGGTATTGGTGCGCCTGAACCTCTTC
2670 2680 2690 2700 2710 2720 2730 2740 2750 2760 2770
p53 related factors
KLF4 group
KLF6
FPM315
BRCA1
muscle initiator
CCTCCTCCCTTTATGTTTGCGTTGCTTTACTCCCACCTTTCCTACTTTCCCACTCGTCTCTCGGGTTACCGAATCCCTCCTACACGCCCCTGCCCTGGCTGGCGTGTTTCT
2780 2790 2800 2810 2820 2830 2840 2850 2860 2870 2880
ING4
TGGTGGGTTCCGCTCGCAGGCCGGGTCTGCATGTTCGT
2890 2900 2910 2920 2930 2940 2950 2960 2970 2980 2990

b

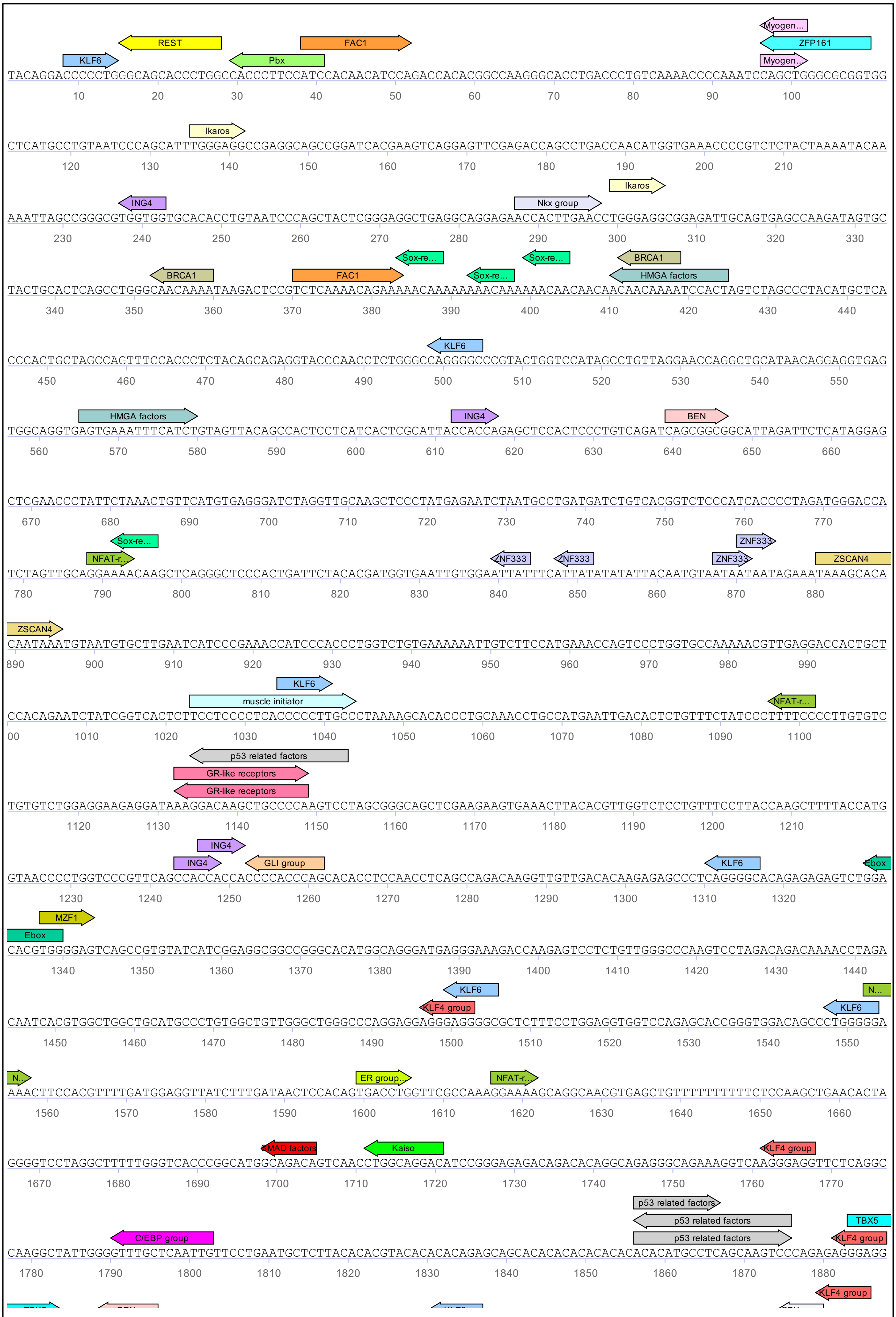

Myogen...
ZFP161
REST
FAC1
KLF6
Pbx
Myogen...
TACAGGACCCCCTGGGCAGCACCCTGGCCACCCTTCCATCCACAACATCCAGACCACACGGCCAAGGGCACCTGACCCTGTCAAAACCCCAAATCCAGCTGGGCGCGGTGG
Ikaros
CTCATGCCTGTAATCCCAGCATTTGGGAGGCCGAGGCAGCCGGATCACGAAGTCAGGAGTTCGAGACCAGCCTGACCAACATGGTGAAACCCCGTCTCTACTAAAATACAA
Ikaros
ING4
Nkx group
AAATTAGCCGGGCGTGGTGGTGCACACCTGTAATCCCAGCTACTCGGGAGGCTGAGGCAGGAGAACCACTTGAACCTGGGAGGCGGAGATTGCAGTGAGCCAAGATAGTGC
Sox-re...
Sox-re...
BRCA1
BRCA1
FAC1
Sox-re...
HMGA factors
TACTGCACTCAGCCTGGGCAACAAAATAAGACTCCGTCTCAAAACAGAAAACAAAAAAAACAAAAAACAACAACAACAACAAAATCCACTAGTCTAGCCCTACATGCTCA
KLF6
CCCACTGCTAGCCAGTTTCCACCCTCTACAGCAGAGGTACCCAACCTCTGGGCCAGGGGCCCGTACTGGTCCATAGCCTGTTAGGAACCAGGCTGCATAACAGGAGGTGAG
HMGA factors
ING4
BEN
TGGCAGGTGAGTGAAATTTCATCTGTAGTTACAGCCACTCCTCATCACTCGCATTACCACCAGAGCTCCACTCCCTGTCAGATCAGCGGCGGCATTAGATTCTCATAGGAG
CTCGAACCCTATTCTAAACTGTTCATGTGAGGGATCTAGGTTGCAAGCTCCCTATGAGAATCTAATGCCTGATGATCTGTCACGGTCTCCCATCACCCCTAGATGGGACCA
Sox-re...
ZNF333
NFAT-r...
ZNF333
ZNF333
ZNF333
ZSCAN4
TCTAGTTGCAGGAAAACAAGCTCAGGGCTCCCACTGATTCTACACGATGGTGAATTGTGGAATTATTTCATTATATATATTACAATGTAATAATAATAGAAATAAAGCACA
ZSCAN4
CAATAAATGTAATGTGCTTGAATCATCCCGAAACCATCCCACCCTGGTCTGTGAAAAAATTGTCTTCCATGAAACCAGTCCCTGGTGCCAAAAACGTTGAGGACCACTGCT
KLF6
muscle initiator
NFAT-r...
CCACAGAATCTATCGGTCACTCTTCCTCCCCTCACCCCCTTGCCCTAAAAGCACACCCTGCAAACCTGCCATGAATTGACACTCTGTTTCTATCCCTTTTCCCCTTGTGTC
p53 related factors
GR-like receptors
GR-like receptors
TGTGTCTGGAGGAAGAGGATAAAGGACAAGCTGCCCCAAGTCCTAGCGGGCAGCTCGAAGAAGTGAAACTTACACGTTGGTCTCCTGTTTCCTTACCAAGCTTTTACCATG
ING4
ING4
GLI group
KLF6
Ebox
GTAACCCCTGGTCCCGTTCAGCCACCACCACCCCACCCAGCACACCTCCAACCTCAGCCAGACAAGGTTGTTGACACAAGAGAGCCCTCAGGGGCACAGAGAGAGTCTGGA
MZF1
Ebox
CACGTGGGGAGTCAGCCGTGTATCATCGGAGGCGGCCGGGCACATGGCAGGGATGAGGGAAAGACCAAGAGTCCTCTGTTGGGCCCAAGTCCTAGACAGACAAAACCTAGA
KLF6
N...
KLF4 group
KLF6
CAATCACGTGGCTGGCTGCATGCCCTGTGGCTGTTGGGCTGGGCCCAGGAGGAGGGAGGGGGCGCTCTTTCCTGGAGGTGGTCCAGAGCACCGGGTGGACAGCCCTGGGGGA
N...
ER group...
NFAT-r...
AAACTTCCACGTTTTGATGGAGGTTATCTTTGATAACTCCACAGTGACCTGGTTCGCCAAAGGAAAAGCAGGCAACGTGAGCTGTTTTTTTTTTCTCCAAGCTGAACACTA
SMAD factors
Kaiso
KLF4 group
GGGGTCCTAGGCTTTTTGGGTCACCCGGCATGGCAGACAGTCAACCTGGCAGGACATCCGGGAGAGACAGACACAGGCAGAGGGCAGAAAGGTCAAGGGAGGTTCTCAGGC
p53 related factors
p53 related factors
TBX5
C/EBP group
p53 related factors
KLF4 group
CAAGGCTATTGGGGTTTGCTCAATTGTTCCTGAATGCTCTTACACACGTACACACAGAGCAGCACACACACACACACACATGCCTCAGCAAGTCCCAGAGAGGGAGG
KLF4 group

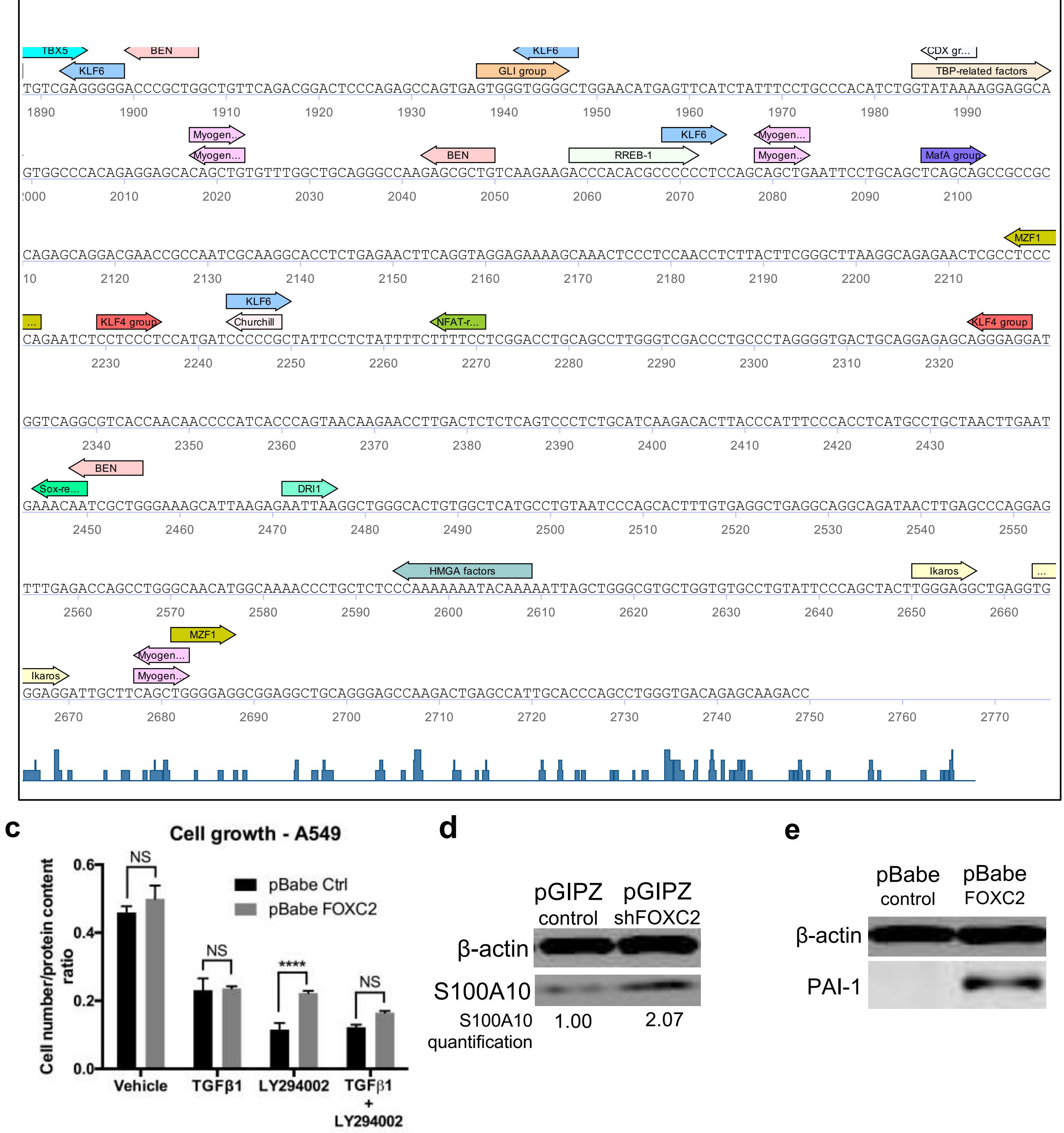

Supplemental figure 4. FOXC2 represses S100A10 expression potentially through direct binding to promoter. Related to figure 3. TRANSFAC analysis of all transcription factor binding sites in the S100A10 (a) and SERPINE1 (PAI-1) (b) promoter regions (2000bp upstream and 1000bp downstream of transcription start site). (c) Quantification of cell growth in A549 pBabe control and pBabe FOXC2 cells treated with TGFβ1 and/or LY294002 after 72 hours. (d) Western blot analysis of S100A10 in A549 cells subject to stable transfection with pGIPZ control or pGIPZ shFOXC2. (d) western blot analysis of PAI-1 in A549 pBabe control and pBabe FOXC2.

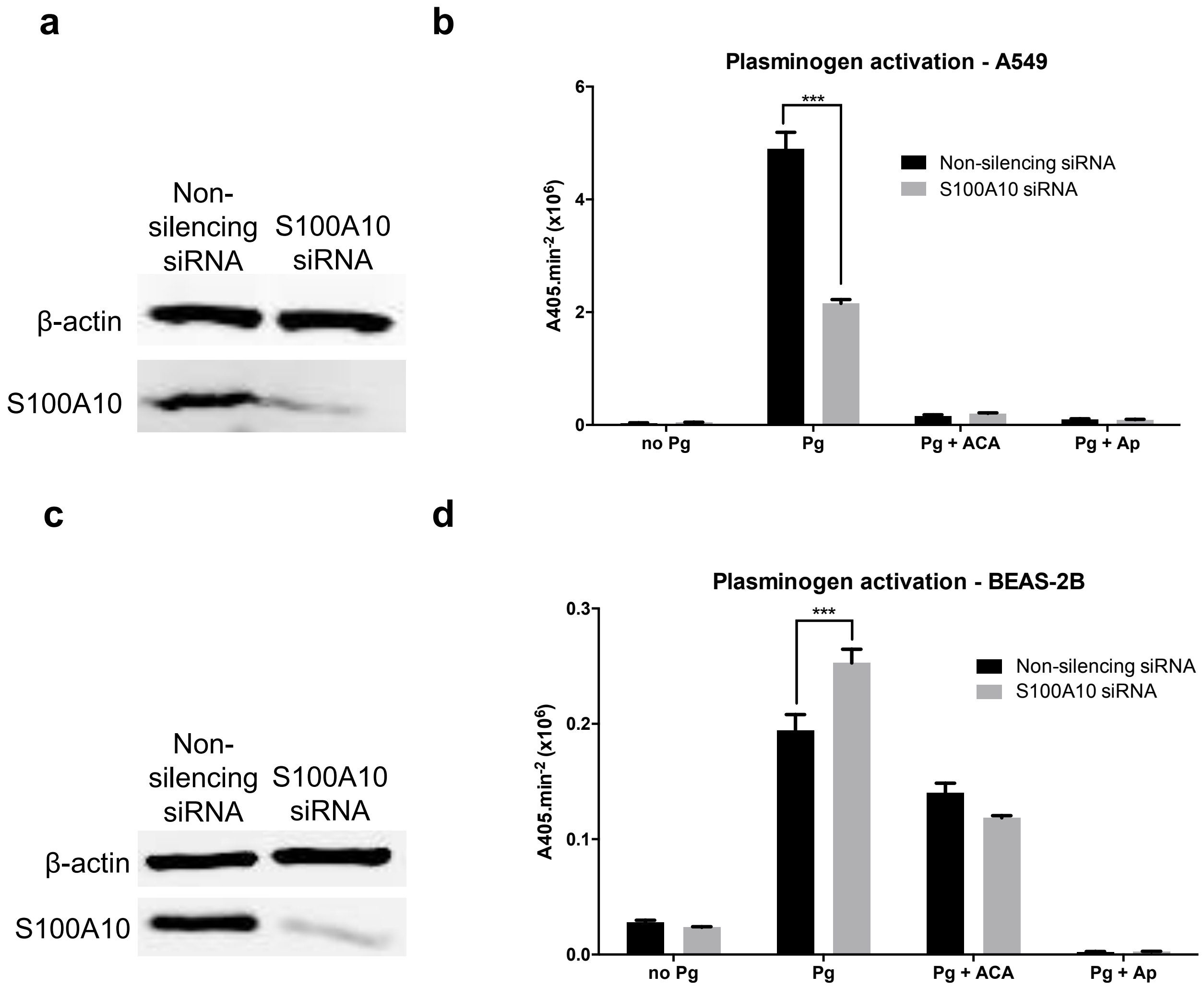

Supplemental figure 5. S100A10 siRNA depletion reduces plasminogen activation at the cell surface of A549 but not BEAS-2B cells. Related to figure 5. Western blot analysis of A549 (a) and BEAS-2B (c) cells which were transiently transfected with non-silencing siRNA or S100A10 siRNA. Plasminogen activation assay of A549 (b) and BEAS-2B (d) cells transfected with non-silencing siRNA and S100A10 siRNA.

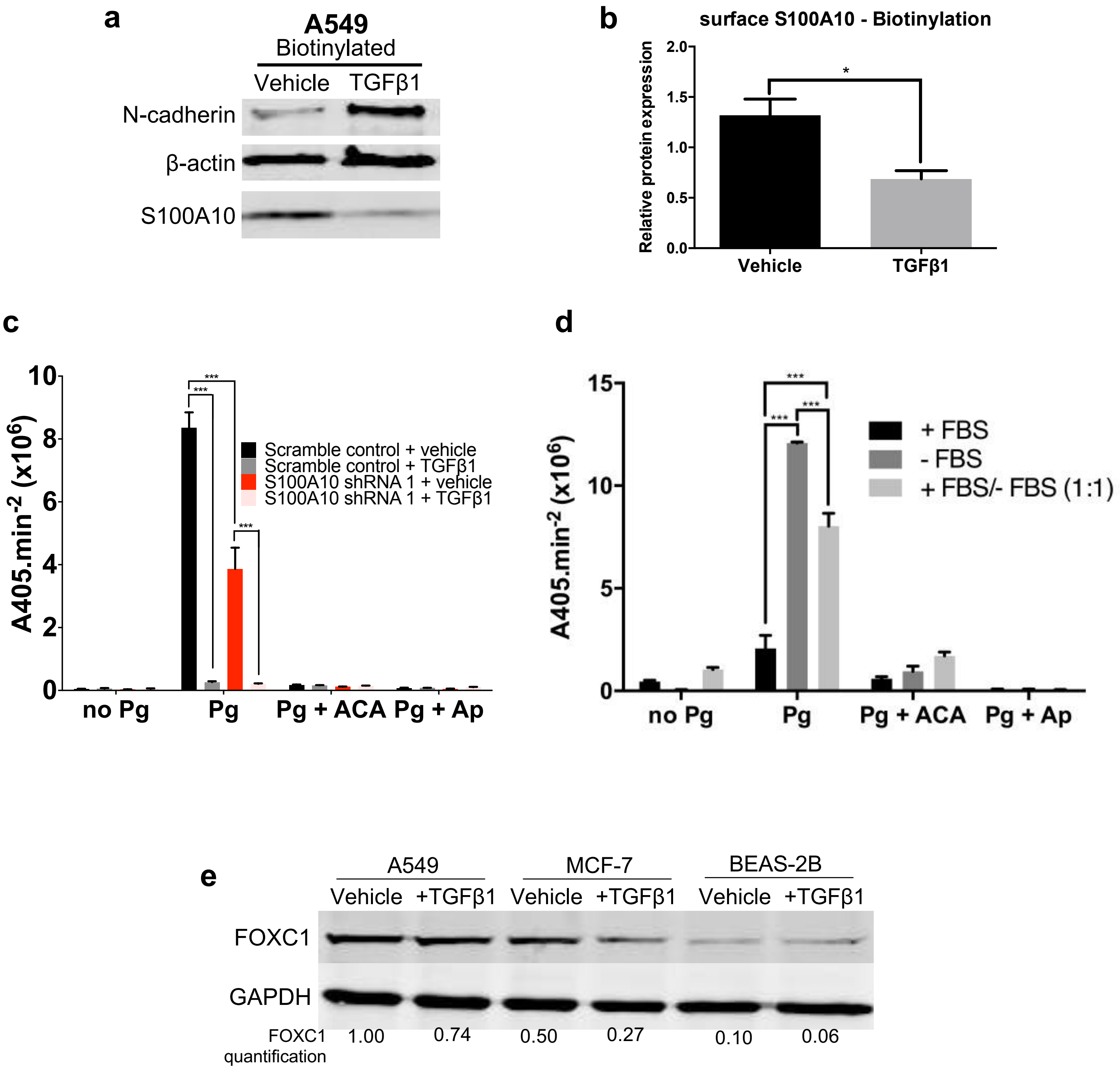

Supplemental figure 6. TGFβ1 treatment and serum modulate plasminogen activation in A549 and BEAS-2B cells respectively partly due reduced cell surface expression of S100A10. Related to figure 6. Western blot analysis (a) and quantification (b) of S100A10 expression in biotinylated lysates from vehicle- or TGFβ1-treated A549 cells. (c) Plasminogen activation of A549 cells with scramble control or S100A10 shRNA 1 and treated with vehicle or TGFβ1. (d) Plasminogen activation of BEAS-2B cells in the presence or absence serum (FBS) or an 1:1 ratio of serum-free and serum-supplemented cells. (e) Western blot analysis and quantification of FOXC1 in A549, MCF-7 and BEAS-2B cells treated with TGFβ1.

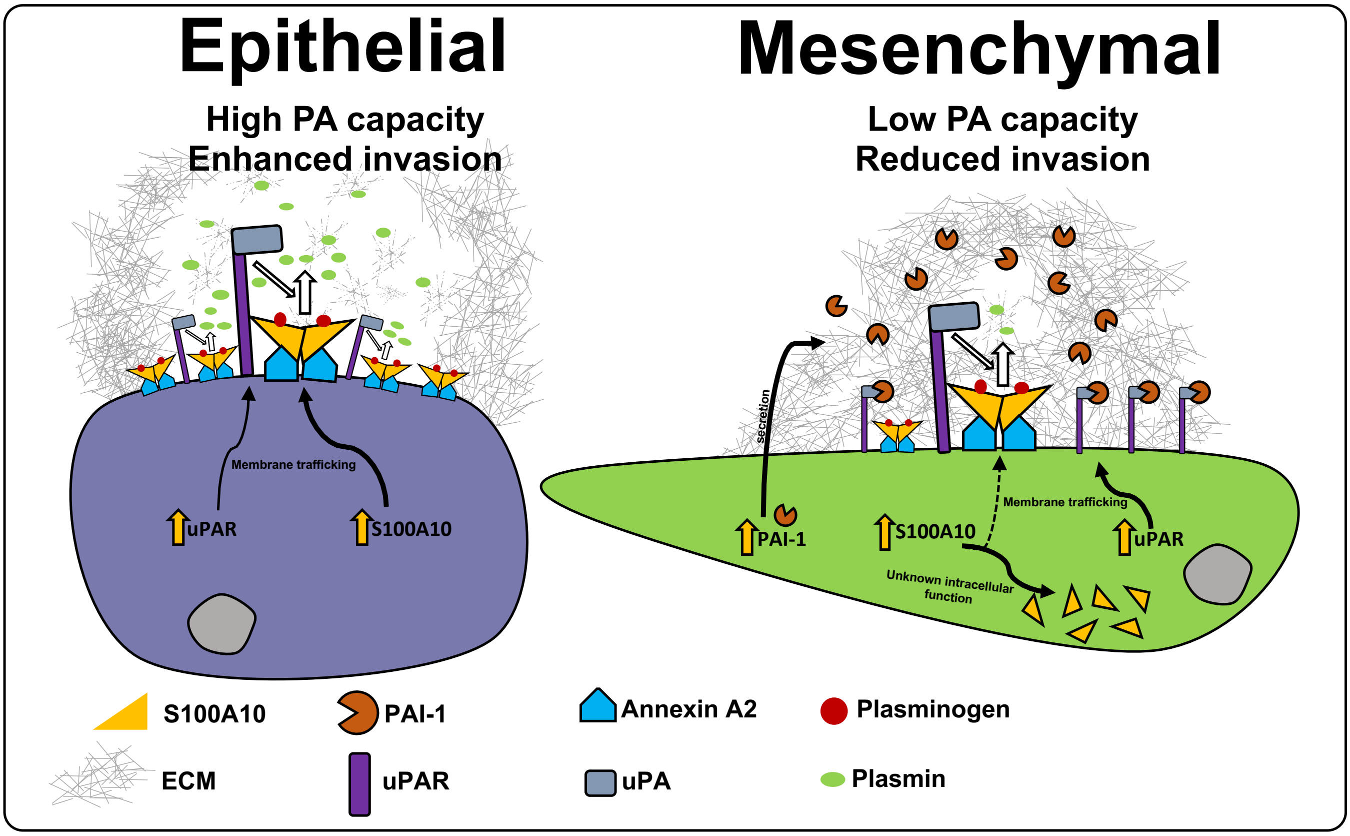

Supplemental figure 7. Proposed model of plasminogen activation at the cell surface of epithelial and mesenchymal cells. Related to figure 8. Although mesenchymal cells upregulate total S100A10 expression, epithelial cells express higher surface levels of S100A10 compared to mesenchymal cells. The latter are likely shuttling S100A10 for an unknown intracellular function. Similarly, both uPAR and PAI-1 are also upregulated in mesenchymal cells. PAI-1 release hinders plasminogen activation into plasmin by inhibiting uPAR-bound uPA. The decrease of plasmin generation reduces extracellular matrix (ECM) degradation. Noteworthy, S100A10 is expressed on the cell surface as part of the annexin A2-S100A10 heterotetramer.

	Significant?	P value	Mean1	Mean2	Difference	SE of difference	t ratio	df
ACTB		0.0655417	1.06665	1.88338	-0.816729	0.32444	2.51735	4
ANXA2		0.656983	140.376	151.382	-11.006	22.9796	0.478947	4
CTSA		0.132142	1886.19	1074.68	811.511	429.955	1.88743	4
CTSB	*	0.00804488	2572.34	5718.74	-3146.41	642.144	4.89985	4
CTSC		0.260814	9360.06	7389.73	1970.32	1505.76	1.30853	4
CTSD	*	4.50158E-05	16731.6	2174.22	14557.3	765.378	19.0198	4
CTSE		0.369556	193396	243732	-50336.3	49829.3	1.01017	4
CTSF		0.134388	117314	75885	41429.3	22121.1	1.87285	4
CTSG		0.412099	267066	208433	58633.1	64100.8	0.914702	4
CTSH		0.116163	72219.2	42703.8	29515.3	14760.2	1.99965	4
CTSK	*	0.00518956	53672.3	92980.4	-39308.1	7094.97	5.54028	4
CTSL		0.0620989	303.94	192.236	111.704	43.4954	2.56817	4
CTSO		0.0850339	197375	130735	66640.8	29262.3	2.27736	4
CTSS	*	0.00305958	96691.7	9001.16	87690.5	13700	6.40077	4
CTSV		0.917622	52691.4	54063.5	-1372.08	12460.5	0.110114	4
CTSW		0.637492	291693	332518	-40825	80193.1	0.509083	4
CTSZ		0.111297	194861	125575	69286.4	34010.5	2.0372	4
ENO1		0.0814024	3106.09	2283.33	822.756	355.092	2.31702	4
HIST1H2BE		0.757777	4258.15	4655.26	-397.108	1202.41	0.330259	4
HMGB1		0.344148	116.294	193.952	-77.6581	72.4519	1.07186	4
ITGA2B		0.598057	323544	343787	-20242.3	35401.1	0.571799	4
ITGAM		0.0890787	290975	193003	97972.1	43828.2	2.23537	4
ITGB2		0.493036	278371	319138	-40767.2	54098.9	0.753569	4
ITGB3		0.407994	239912	270401	-30488.4	33012.5	0.92354	4
KRT8	*	0.0245433	9.70558	4.25953	5.44606	1.54903	3.51578	4
MMP1	*	0.00469768	66499.3	373558	-307059	53920.4	5.69467	4
MMP10	*	0.00541865	9846.51	462807	-452961	82742.1	5.47437	4
MMP11		0.269333	380369	275004	105365	82234.3	1.28127	4
MMP12		0.45227	297901	339352	-41450.5	49829.2	0.831851	4
MMP13		0.339444	535358	697862	-162503	149950	1.08372	4
MMP14		0.482827	195934	210831	-14897	19279.4	0.772689	4
MMP15		0.945628	289955	284156	5799.18	79904.7	0.0725762	4
MMP16		0.827978	409330	397835	11494.8	49562.4	0.231925	4
MMP17		0.361487	332274	258496	73778.8	71675.7	1.02934	4
MMP19		0.055105	266018	200290	65728	24505.6	2.68216	4
MMP2	*	0.000152514	112405	288096	-175692	12581	13.9648	4
MMP20		0.853672	307686	296120	11566	58808.6	0.196672	4
MMP21		0.839517	425599	409984	15615.2	72274.9	0.216053	4
MMP23A/23B		0.259226	515981	331526	184454	140408	1.3137	4
MMP24	*	0.00589544	194384	83372.1	111012	20759.6	5.34752	4

MMP24-AS1		0.119836	42212.9	31079.9	11133.1	5644.36	1.97243	4
MMP25		0.305215	230548	207671	22877.1	19472.2	1.17486	4
MMP26		0.311743	421357	268669	152687	131994	1.15677	4
MMP27		0.610768	225605	244811	-19206.6	34838.1	0.551309	4
MMP28		0.159793	228389	281823	-53433.3	30993.1	1.72404	4
MMP3	*	0.0395219	245540	321228	-75687.8	25139.3	3.01073	4
MMP7	*	0.00348635	199413	238.3	199175	32237.4	6.17838	4
MMP8		0.264265	367913	313873	54039.8	41652.9	1.29739	4
MMP9		0.173403	125029	174036	-49007.8	29623.9	1.65433	4
PLAT	*	0.00501131	59076.7	327208	-268131	47931.2	5.59408	4
PLAU		0.0622362	114.606	10509	-10394.4	4050.67	2.56608	4
PLAUR	*	0.00604463	2644.46	25480.7	-22836.3	4300.3	5.31039	4
PLG		0.761346	390284	362913	27371.1	84173.3	0.325175	4
PLGRKT		0.0596164	16554.7	8881.24	7673.5	2943.58	2.60686	4
RUVBL1		0.0613583	6309.61	4839.27	1470.35	570.007	2.57952	4
S100A10	*	0.000508743	83.6273	423.151	-339.523	33.091	10.2603	4
S100A4	*	0.021085	30.8968	6.87697	24.0199	6.51579	3.68641	4
SERPINA1		0.977307	72287.8	73351.7	-1063.87	35154.1	0.0302631	4
SERPINA10		0.437131	408445	316902	91543.4	106157	0.862344	4
SERPINA12		0.177996	209476	275862	-66386.1	40675.4	1.6321	4
SERPINA2		0.542926	410246	443578	-33332.2	50188.3	0.664143	4
SERPINA3		0.0729967	358348	204801	153547	63527	2.41704	4
SERPINA4		0.0833094	279671	206226	73444.7	31988.8	2.29595	4
SERPINA5		0.42862	215158	277864	-62706.4	71268.4	0.879863	4
SERPINA6	*	0.000446666	399969	47314.7	352655	33237.6	10.6101	4
SERPINA7		0.30982	322074	227708	94365.8	81205.2	1.16207	4
SERPINA9	*	0.0180716	64698.5	123471	-58772.9	15206.8	3.8649	4
SERPINB1	*	1.95894E-05	61986.9	5475.71	56511.2	2409.43	23.4542	4
SERPINB10		0.856401	312501	326968	-14467.9	74983.7	0.192947	4
SERPINB11		0.505211	364216	324753	39463.4	53974.3	0.731153	4
SERPINB12		0.985868	283861	281805	2056.6	109138	0.018844	4
SERPINB13		0.272733	425399	384133	41266.5	32477.2	1.27063	4
SERPINB2		0.820274	494131	461659	32471.7	133870	0.242562	4
SERPINB3	*	0.0151873	352309	207454	144855	35566	4.07285	4
SERPINB3/B4	*	0.010058	246752	130380	116372	25317.9	4.59642	4
SERPINB4		0.126558	449017	311405	137611	71490.6	1.92489	4
SERPINB5		0.866615	448047	466398	-18351.3	102503	0.179032	4
SERPINB6		0.659616	145559	161584	-16024.8	33742.6	0.474914	4
SERPINB7	*	0.00361606	285737	104691	181046	29595.5	6.11735	4
SERPINB8		0.336268	121859	98346.9	23511.7	21534.5	1.09181	4
SERPINB9		0.271465	59337.3	43647.1	15690.2	12310	1.27459	4
SERPINC1		0.557411	339460	310797	28663.4	44836.6	0.639287	4
SERPIND1		0.909029	412118	419110	-6992.77	57474.1	0.121668	4

SERPINE1	*	0.015365	225.648	188463	-188237	46378.5	4.05871	4
SERPINE2	*	0.000295171	409.58	28035.5	-27625.9	2341.13	11.8003	4
SERPINF1		0.131914	376471	302248	74223	39293.7	1.88893	4
SERPINF2	*	0.0396057	222236	161020	61216.6	20347.3	3.00858	4
SERPING1		0.340708	387056	508577	-121521	112465	1.08052	4
SERPINH1		0.165028	9334.56	14130.3	-4795.74	2826.82	1.69651	4
SERPINI1		0.0738383	237296	373100	-135804	56433.3	2.40645	4
SERPINI2		0.61928	319009	307210	11798.8	21941.4	0.53774	4
TIMP1		0.386609	102.239	74.0368	28.2023	29.0497	0.970828	4
TIMP2	*	0.0121503	968.717	27537	-26568.2	6106.64	4.35071	4
TIMP3	*	0.000638519	17834.8	246146	-228311	23597.2	9.67534	4
TIMP4		0.369286	73150.2	51614.7	21535.5	21305.2	1.01081	4
KLK1		0.982762	141113	140693	420.128	18277.1	0.0229866	4
KLK2		0.637777	362861	396745	-33883.9	66617	0.508638	4
KLK3		0.918952	290340	292330	-1990.5	18374.7	0.108329	4
KLK4		0.747751	180908	171971	8937.03	25934.6	0.344599	4
KLK5		0.160476	100580	81143.5	19436.2	11297.6	1.72039	4
KLK6		0.0988511	139809	177246	-37437.4	17476.7	2.14213	4
KLK7		0.969859	201993	200993	999.453	24861	0.0402016	4
KLK8		0.544346	194907	219726	-24818.8	37508.5	0.661685	4
KLK9		0.309813	358755	296180	62575.3	53847.5	1.16208	4
KLK10		0.141366	539383	356994	182390	99712.3	1.82916	4
KLK11		0.28774	262501	355155	-92654.5	75629.4	1.22511	4
KLK12		0.529046	166905	158212	8692.7	12627.4	0.688401	4
KLK13		0.426468	309352	367068	-57716.3	65265	0.884338	4
KLK14	*	0.00230252	313009	202912	110097	15935.8	6.90876	4
KLK15	*	0.0178079	284197	351997	-67799.7	17464.2	3.88221	4

Supplemental Table 1. Gene expression analysis of 130 components of the plasminogen activation system in response to TGFβ1 treatment in A549 cells. These components include plasminogen activators (*PLAU*, *PLAT*), plasminogen activator receptors (*PLAUR*), plasminogen activator inhibitors (e.g. *SERPINE1*), plasminogen receptors (e.g. *ENO1, HMGB1, RUVBL1, S100A10*), MMPs, MMP inhibitors (*TIMPs*) and kallikreins (*KLK*s). The expression data of vehicle-treated and TGFβ1-treated (72-hour time point) cells was obtained from the gene expression omnibus (GEO; access code GSE17708) (Sartor *et al.* 2010). The expression values were first normalized against the expression house-keeping genre EF1A then against a sample with the lowest normalized expression value. The cut-off for the adjusted p-value was 0.05.
